# Ceramide-Induced Apoptosis in Renal Tubular Cells: A Role of Mitochondria and Sphingosine-1-Phoshate

**DOI:** 10.3390/ijms16035076

**Published:** 2015-03-05

**Authors:** Norishi Ueda

**Affiliations:** Department of Pediatrics, Public Central Hospital of Matto Ishikawa, 3-8 Kuramitsu, Hakusan, Ishikawa 924-8588, Japan; E-Mail: nueda@mattohp.com; Tel.: +81-76-275-2222; Fax: +81-76-274-5974

**Keywords:** Bcl-2 family proteins, caspases, ceramide, growth factors, mitochondria, mitogen-activated protein kinases, reactive oxygen species, renal tubular cells, sphingosine-1 phosphate

## Abstract

Ceramide is synthesized upon stimuli, and induces apoptosis in renal tubular cells (RTCs). Sphingosine-1 phosphate (S1P) functions as a survival factor. Thus, the balance of ceramide/S1P determines ceramide-induced apoptosis. Mitochondria play a key role for ceramide-induced apoptosis by altered mitochondrial outer membrane permeability (MOMP). Ceramide enhances oligomerization of pro-apoptotic Bcl-2 family proteins, ceramide channel, and reduces anti-apoptotic Bcl-2 proteins in the MOM. This process alters MOMP, resulting in generation of reactive oxygen species (ROS), cytochrome C release into the cytosol, caspase activation, and apoptosis. Ceramide regulates apoptosis through mitogen-activated protein kinases (MAPKs)-dependent and -independent pathways. Conversely, MAPKs alter ceramide generation by regulating the enzymes involving ceramide metabolism, affecting ceramide-induced apoptosis. Crosstalk between Bcl-2 family proteins, ROS, and many signaling pathways regulates ceramide-induced apoptosis. Growth factors rescue ceramide-induced apoptosis by regulating the enzymes involving ceramide metabolism, S1P, and signaling pathways including MAPKs. This article reviews evidence supporting a role of ceramide for apoptosis and discusses a role of mitochondria, including MOMP, Bcl-2 family proteins, ROS, and signaling pathways, and crosstalk between these factors in the regulation of ceramide-induced apoptosis of RTCs. A balancing role between ceramide and S1P and the strategy for preventing ceramide-induced apoptosis by growth factors are also discussed.

## 1. Introduction

Ceramide, which belongs to sphingolipids, is now recognized as a signaling molecule within the cell, although sphingolipids have long been considered to be only structural components of the cell membranes. Over the last two decades, significant research on sphingolipids has focused on the two distinct natures of ceramide and sphingosine-1-phosphate (S1P), which differentially regulate many cellular signaling pathways [1,2]. This leads to discovery that these sphingolipids play a crucial role in the regulation of apoptosis, cell cycle arrest, proliferation, senescence, stress response, and autophagy. Ceramide typically induces growth arrest and apoptosis [2,3], whereas S1P promotes cell proliferation and survival [1,2]. Other sphingolipids such as sphingosine and sphinganine also function as pro-apoptotic factors [1,2]. Thus, the dynamic balance between ceramide, S1P and these apoptotic sphingolipids play a crucial role in the regulation of ceramide-induced apoptosis.

Mitochondria play a central role in the regulation of ceramide-induced apoptosis through an alteration of mitochondrial outer membrane permeability (MOMP) [4,5]. MOMP is tightly regulated by Bcl-2 family proteins [5], resulting in generation of reactive oxygen species (ROS), the release of cytochrome C into the cytosol, and the initiation of caspase cascade and apoptotic process [4,5].

Besides the mitochondria-mediated cellular events, there are many downstream cellular targets for ceramide in the regulation of apoptosis. One of these is mitogen-activated protein kinases (MAPKs), which regulate ceramide-induced apoptotic process [2]. MAPKs, on the other hand, can regulate ceramide generation by regulating the enzymes involved in ceramide metabolism, thereby affecting ceramide-induced apoptosis [6,7]. Ceramide also regulates other kinases, leading to apoptosis. In addition, there is a crosstalk between Bcl-2 family proteins, ROS, and many signaling molecules including MAPKs, leading to the regulation of ceramide-induced apoptosis. Growth factors can rescue ceramide-induced apoptosis through the regulation of intracellular levels of ceramide and S1P [1], Bcl-2 family proteins [8], MAPKs [8,9], and other signaling molecules, and thus may be a potential therapeutic strategy for ceramide-induced apoptosis.

Ceramide can be generated in renal tubular cells (RTCs) after exposure to various stimuli. However, little is known about a role of ceramide in apoptosis of RTCs and the mechanism by which ceramide regulates the apoptotic processes in RTCs. This review aims to summarize evidence supporting a role of ceramide and the balance between ceramide and other sphingolipids, in particular S1P, in the regulation of apoptosis in RTCs. I will discuss a role of mitochondria, including alteration of MOMP, Bcl-2 family proteins and ROS generation, MAPKs, and other kinases as well as a crosstalk between these factors in the regulation of ceramide-induced apoptosis of RTCs. Strategy for preventing ceramide-induced apoptosis by growth factors, which can regulate ceramide metabolism and MAPKs, will also be discussed.

## 2. Ceramide Biosynthesis and Degrading Pathways in Mammalian Cells

**Figure 1:**
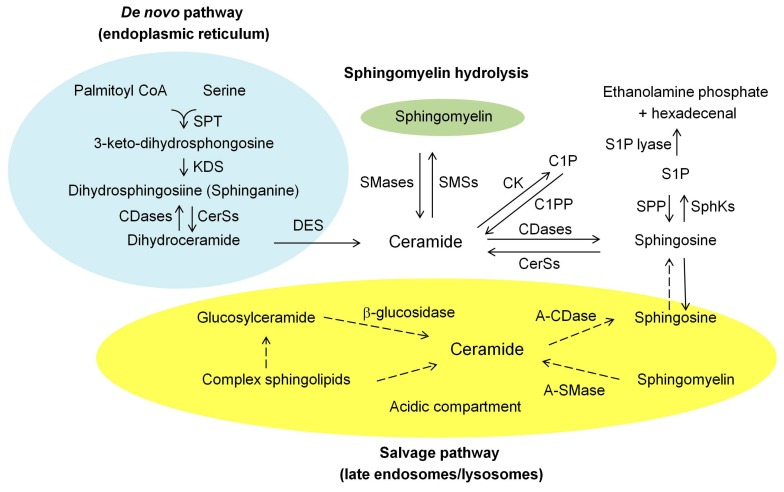
Metabolism of sphingolipids. Ceramide can be generated by three major pathways: (1) the *de novo* synthesis pathway, which occurs in the endoplasmic reticulum; (2) hydrolysis of sphingomyelin; and (3) the salvage pathway, which occurs in acidic compartment of the late endosomes/lysosomes. A-CDase, acid ceramidase; A-SMase, acid sphingomyelinase; CerSs, ceramide synthases; CK, ceramide kinase; C1P, ceramide-1-phosphate; C1PP, C1P phosphatase; DES, dihydroceramide desaturase; KDS, 3-keto-dihydrosphingosine reductase; SMases, sphingomyelinases; SMSs, sphingomyelin synthases; SphKs, sphingosine kinases; S1P, sphingosine-1-phosphate; SPP, S1P phosphatase; SPT, serine palmitoyl transferase.

Ceramide is composed of sphingosine and a fatty acid [3,10]. Upon stimuli, ceramide can be generated within the cell through the three major pathways (Figure 1): (1) *de novo* synthesis mediated by ceramide synthases (CerSs); (2) hydrolysis of sphingomyelin (SM) by sphingomyelinases (SMases); and (3) the recycling or salvage pathway [3.10].

### 2.1. De Novo Synthesis Pathway

The *de novo* synthesis pathway is the best characterized ceramide-generating pathway, which mainly occurs in the endoplasmic reticulum (ER) and to a lesser extent the mitochondrial membrane [3,10] (Figure 1). This pathway begins with the condensation of amino acid l-serine and palmitoyl-CoA, which is catalyzed by serine palmitoyl transferase (SPT) to form 3-keto-dihydrosphingosine (3-keto-dihydro-Sph) [2,3,10]. 3-keto-dihydro-Sph is subsequently reduced to form dihydrosphingosine (sphinganine) mediated by an action of 3-keto-dihydro-Sph reductase. Dihydrosphingosine is then acylated by CerSs to form dihydroceramide. In mammals, there are six isoforms of CerSs (CerS1-6), which show substrate preference for specific chain-length fatty acyl CoAs [2]. Dihydroceramide is subsequently desaturated by dihydroceramide desaturase [3,10], generating ceramide. Once generated, ceramide may amass or be converted to various metabolites.

### 2.2. Hydrolysis of the Sphingomyelin (SM) Pathway

The second ceramide-generating pathway involves the hydrolysis of SM, which occurs in the plasma membranes, lysosomes, ER, Golgi, and mitochondria [3,10]. This process is mediated by either acid sphingomyelinase (A-SMase) or neutral sphingomyelinases (N-SMases), generating ceramide and phosphocholine [2,3,10] (Figure 1). The SMases have multiplicity, their own pH optima, and distinct subcellular localization [2,3,10]. SM is the most abundant sphingolipid, and thus it is an enormous source of ceramide generation within the cell.

### 2.3. Salvage Pathway

A more complex regulation of intracellular ceramide levels is the salvage pathway [2,3,10] (Figure 1). This pathway involves the recycling of sphingosine that is produced by the breakdown of sphingolipids and glycosphingolipids (GSLs), and occurs in the acidic subcellular compartments of the lysosomes and/or the late endosomes [2,3,10]. Many enzymes are involved in this pathway, including A-SMase, glucocerebrosidase (acid β-glucosidase), acid ceramidase (A-CDase) and CerSs. SM is cleaved by A-SMase to form ceramide. Additionally, the breakdown of GSLs through sequential removal of their terminal hydrophilic portions catalyzed by specific hydrolases leads to the formation of glucosylceramide and galactosylceramide, which are subsequently hydrolyzed by acid β-glucosidases and galactosidase, respectively, generating ceramide [2,3,10]. Then, the common metabolic product, ceramide, generated by either pathway is further deacylated by A-CDase to generate sphingosine and free fatty acid that can leave the lysosomes and enter into the cytosol [2,3,10]. Once entered into the cytosol, the released sphingosine may re-enter the pathways for the synthesis of ceramide and/or S1P and becomes as a substrate. The salvage pathway re-utilizes sphingosine to form ceramide by an action of CerSs [2,3,10]. Recently, CerS5 and CerS6 have been shown to be involved in the salvaging pathway [11]. The released sphingosine is also phosphorylated by sphingosine kinases (SphKs) to form S1P [1], which in turn can be dephosphorylated by S1P phosphatases, regenerating sphingosine [2,3,10]. S1P is finally metabolized by S1P lyase to release ethanolamine phosphate and hexadecenal [2,3]. The salvage pathway may account for more than a half of the sphingolipid biosynthesis within the cell [10].

### 2.4. Degrading Pathway

Ceramide is metabolized by phosphorylation via ceramide kinase to form ceramide-1 phosphate (C1P), which can be recycled by C1P phosphatase [2,3] (Figure 1). Ceramide is deacylated by either A-CDase or N-CDase to form sphingosine, which may be converted back to ceramide via CerSs, or phosphorylated by SphKs to form S1P [2,3]. Ceramide can be converted back to SM by transfer of phosphorylcholine from phosphatidylcholine to ceramide via SM synthases (SMSs), and glycosylated by glucosylceramide synthase to form glucosylceramide [12]. Thus, the metabolism of sphingolipids involves the more complex biosynthetic and degrading pathways, and ceramide is not only a central sphingolipid metabolite but also a “hub” of sphingolipid metabolism, being served as either product or substrate [3].

## 3. Compartmentalization of Ceramide Metabolism and Trafficking of Ceramide 

Ceramide metabolism is restricted to cellular membranes and highly compartmentalized since many ceramides found in mammalian cellular membranes contain long fatty acyl chains of 16–28 carbon atoms, rendering them hydrophobic lipids [3]. Ceramide, which can be generated by the membrane-associated enzymes, exerts its biological effects either in close proximity to the generation site or requires specific transport mechanisms to reach its cellular targets in another intracellular compartment [2,3]. In this section, the localization of the enzymes involved in ceramide metabolism, ceramide compartmentalization, and trafficking of ceramide within the cells, including RTCs, are discussed.

### 3.1. Intracellular Localization of the Enzymes Involved in Ceramide Metabolism

In mammals, 6 CerSs, at least 4 or 5 SMases, and 5 CDases have been identified, and they have distinct intracellular localization [3]. CerSs reside in the ER [3]. CerS1 and CerS6 show perinuclear localization, and the former can translocate into the Golgi [3]. CerS2, 4, and 6 are also localized in the MOM and mitochondrial inner membrane (MIM) [4,13]. HeLa cells do not have mitochondrial CerSs [14], suggesting that mitochondrial localization of CerSs vary with cell or tissue types. SMases and CDases have distinct subcellular localization such as the ER, Golgi, mitochondria, lysosomes, and plasma membrane [3]. Recently, N-SMase [15], which hydrolyses SM into ceramide, and N-CDase [16], which catalyses condensation of palmitate and sphingosine into ceramide, have been identified in murine and rat mitochondria, respectively. Other enzymes involved in ceramide metabolism such as SMSs (SMS1 and 2) reside in the plasma membrane and Golgi [17], and glucocerebrosidases are localized in the lysosomes [12]. The nuclei contain different species of sphingolipids, including SM (the most abundant nuclear sphingolipid), ceramide, sphingosine, and gangliosides, and the enzymes involved in sphingolipid metabolism such as SMase, SMS, SphK1, ceramide kinase, and CDases [18]. Nuclear SM breakdown by N-SMase results in increased nuclear ceramide levels, and/or sphingosine accumulation by an action of nuclear CDases, and sphingosine can be converted to S1P by Sphk2 in the nuclei [18].

SphKs phosphorylate sphingosine to form S1P. SphK1 resides mainly in the cytosol, and some associate with the plasma membrane [1]. In contrast, SphK2 resides in the cytosol, plasma membrane, mitochondria, and nucleus, and the subcellular localization of SphKs depends on the cell types. Thus, distinct subcellular localization of the enzymes involved in sphingolipid metabolism contributes to high compartmentalization of ceramide within the cell.

### 3.2. Ceramide Compartmentalization and Trafficking

#### 3.2.1. Endoplasmic Reticulum (ER) and Golgi

**Figure 2:**
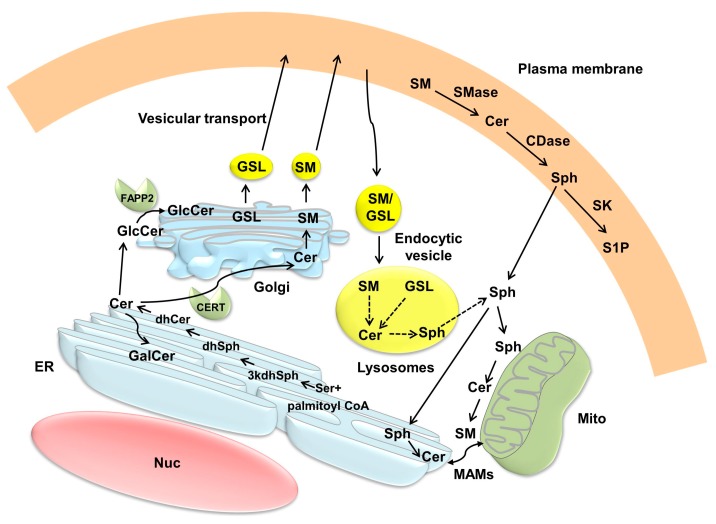
Compartmentalization and trafficking of ceramide. Ceramide (Cer) is synthesized *de novo* on the cytosolic surface of the endoplasmic reticulum (ER), and then transported via ceramide transport protein (CERT) to the Golgi, where it becomes as a substrate for sphingomyelin (SM) synthesis. Cer in the ER can also be transferred to the Golgi by vesicular transport, and subsequently the transport protein, four-phosphate adaptor protein 2 (FAPP2), delivers glucosylceramide (GlcCer) as a precursor for the synthesis of glycosphingolipids (GSL). SM and GSL are then transported to the plasma membrane via vesicular transport. SM and GSL also move from the plasma membrane to the endosomal system and are metabolized via lysosomal degradation. Ceramide is transformed to galactosylceramide (GalCer) in the ER. Ceramide can be exchanged between the ER and mitochondria through mitochondria-associated membranes (MAMs) at the ER. Because of its adequate solubility, sphingosine can leave the lysosome and move between membranes. CDase, ceramidase; dhCer, dihydroceramide; dhSph, dihydrosphingosine; 3kdhSph, 3-keto-dihydrosphingosine; Mito, mitochondria; Nuc, nucleus; Ser, serine; SK, sphingosine kinase; Sph, sphingosine; S1P, sphingosine-1-phosphate.

*De novo* ceramide synthesis occurs in the ER [2,3] (Section 2.1, Figure 1 and Figure 2). Once formed in the ER, ceramide can be transported to the Golgi by either vesicular trafficking or by non-vesicular trafficking mediated by the proteins such as ceramide transport protein, CERT [2,3,12] (Figure 2). Once transferred, ceramide is further metabolized to SM and glucosylceramide in the Golgi, with the latter compound being as the precursor for complex GSLs. Phosphorylation of CERT at the phenylalanines in an acidic tract motif-adjacent serine, which associates with the ER-resident membrane protein, vesicle-associated membrane protein-associated protein (VAP), can enhance the interaction of CERT with VAP, thereby regulating the inter-organelle trafficking of ceramide in response to the perturbation of intracellular SM and/or other sphingolipids [19]. Ceramide can also move from the ER via vesicular transport, and then the glucocylceramide transfer protein, four-phosphate adaptor protein 2 (FAPP2), delivers glucosylceramide as precursor for GSL synthesis across the Golgi network [2,3]. S1P phosphohydrolase-1 located mainly in the ER can increase ceramide levels in the ER by inhibiting the anterograde membrane transport of ceramide from the ER to the Golgi [20].

#### 3.2.2. Mitochondria

Mitochondria-associated membranes (MAMs) are distinct membranes at the ER, which closely contact with mitochondria, and facilitate the exchange of lipids and lipid-derived molecules such as ceramide between the two organelles [21]. By this mechanism, *de novo* generated ceramide in the ER can be transferred to mitochondria [21]. In addition, ceramide generated at the plasma membrane upon stimuli in turn self-associates into the platforms, and is subsequently invaginated and fused with mitochondria, facilitating a direct transfer of ceramide from the plasma membrane to the MOM [22]. Mitochondria, which contain sphingolipids such as SM and ceramide, are not only an important intracellular compartment for ceramide metabolism [13,16] but also act as “sensors” of cellular stress by sensing the local accumulation of specific sphingolipids and glycolipids [23].

#### 3.2.3. Plasma Membrane and Lysosomes/Endosomes

Ceramide can be generated at the plasma membrane by an action of SMases and possibly neutral glucocerebrosidase that resides in the lysosomes and mitochondria, resulting in compartment-specific ceramide formation [3]. Ceramide can be also phosphorylated at the plasma membrane by ceramide kinase to form C1P, which in turn is dephosphorylated by an action of C1P phosphatase, generating ceramide [2,3]. In the salvage pathway, the sphingosine released into the cytosol can be re-acylated to form ceramide in the late endosomes and lysosomes [3,10]. Because of its ionaizable positive charge and adequate solubility, sphingosine can leave the lysosomes and move between membranes [2,3]. Upon apoptotic stimuli, translocation of A-SMase in the lysosomes to the plasma membrane occurs through lysosome exocytosis, resulting in the formation of ceramide-enriched membrane platforms in the plasma membrane [24].

#### 3.2.4. Nuclei

The nuclei contain N-SMase, SMS, ceramide kinase, and CDases, suggesting that ceramide is actively produced and consumed in the nuclei (reviewed in [18]). Nuclear SM breakdown by N-SMase, resulting in increased nuclear ceramide levels, and/or sphingosine accumulation via an action of CDase occur in the nuclei. Taken together, ceramide metabolism is highly compartmentalized, and because of limited capacity of ceramide for intracellular diffusion, trafficking of ceramide by vesicular or non-vesicular ways plays an important role in the regulation of ceramide-induced apoptosis [2,3].

### 3.3. Ceramide Compartmentalization and Trafficking in Renal Tubular Cells (RTCs)

A role of ceramide compartmentalization and trafficking in the regulation of ceramide-induced apoptosis of RTCs has not been completely revealed. Ceramide is specifically distributed to the Golgi compartment at the base of the primary cilium of Madin–Dabry canine kidney (MDCK) cells [25]. In these cells, ceramide can be generated in the endosomes by the salvage pathway [26], through which A-SMase can hydrolyze the membrane-bound and endocytosed SM, leading to formation of ceramide [10]. This process is critical for the distribution of SM and ceramide to the apical membrane of MDCK cells. The SM is rich at the apical membrane of MDCK cells, but when expressed intracellularly, SM is found in the Golgi but not in the nucleus, mitochondria, ER or plasma membrane [27]. The content of SM on the outer leaflet of the plasma membrane can inhibit the transbilayer movement of diacylglycerol into the plasma membrane, which is a key component in lipid metabolism and signaling in MDCK cells [28]. In baby hamster kidney cells, C6-NBD-glucosylceramide localized in the endosomes can move into the plasma membrane and the Golgi, where it is further recycled [29].

The enzymes involved in ceramide metabolism reside in the kidney, including CerSs [30,21,32,33,34], CDases [35,36], N-SMases [36] and SphKs [37]. CerSs [38], including CerS2 [34] and CerS6 [30], reside in the microsomes of RTCs. Apoptotic stimuli such as ultraviolet (UV) light can induce translocation of CerS1 from the ER to the Golgi apparatus in human embryonic kidney (HEK)293 cells [39] (Table 1). N-CDase is mainly localized in the apical membrane, ER and Golgi of RTCs [35]. N-SMase1 is abundant in the kidney, and localized in the ER and Golgi, but not in the plasma membrane of HEK293 cells [40] A-SMase is exclusively localized in the lysosomes, whereas most, if not all, N-SMase resides in the microsomes and plasma membrane of human RTCs [41]. SphKs and S1P receptors (S1PRs) are expressed in the kidney [42]. Both SPhK1 and SPhK2 are expressed in glomerular mesangial cells [43], human kidneys and RTCs [37,44,45]. Upon stimuli, SphK1 in the cytosol can move to the plasma membrane [46], and SphK2, which resides in the plasma membrane and to a lesser extent in the cytosol, is translocated to the ER and Golgi in HEK293 cells [47].

## 4. Ceramide-Induced Apoptosis

### 4.1. Overview of Apoptosis

Apoptosis is a cell suicide program that regulates development, tissue homeostasis, immunity, and many pathological conditions [48]. There are two distinct apoptotic pathways; extrinsic (death receptor-mediated) and intrinsic pathways (mitochondria-mediated). The extrinsic pathway involves death receptor pathway that is triggered by binding of ligands such as FasL or tumor necrosis factor (TNF)-α to a death receptor on the plasma membrane, which in turn activates the initiator caspase-8 through the formation of the death-inducing signaling complex, consisting of a death receptor and a death domain-containing adaptor protein [48]. Its activation proteolytically cleaves and activates the effector caspases (e.g., caspase-3, -6, and -7), which function as downstream effectors of apoptosis [48]. Caspases are a family of cysteine proteases that cleave many cellular targets, resulting in characteristic morphology and DNA change, and ultimately phagocytic removal of the apoptotic cells [48].

The intrinsic pathway can be triggered by various cellular stresses, which converge at the mitochondria. Upon apoptotic stimuli, the Bcl-2 family proteins encoded by a gene called B-cell lymphoma 2 (BCL-2) are activated and translocated into the MOM, which differentially regulate the MOMP [49]. The balance between the pro- and anti-apoptotic members of the Bcl-2 family proteins tightly regulates the mitochondrial integrity. Increased MOMP allows transport of apoptogenic proteins in the mitochondrial intermembrane space into the cytosol, including cytochrome C, Smac and Omi that bind to and inhibit the inhibitor of apoptosis proteins (IAP), apoptosis inducing factor (AIF) and endonuclease G, initiating a caspase cascade. Binding of the released cytosolic cytochrome C to the adaptor protein, apoptosis protease-activating factor-1 (APF-1) and procaspase-9 forms a complex, termed “apoptosome complex”. Within the apoptosome complex, the executional caspase-9 is activated, leading to processing caspase-3 and initiation of apoptosis.

### 4.2. Ceramide-Induced Apoptosis in RTCs

Under normal condition, intracellular ceramide levels are maintained low by its rapid conversion into less deleterious sphingolipids. However, pathological conditions disturb ceramide metabolism, leading to accumulation of ceramide. Ceramide is generated and accumulated in RTCs in response to various stimuli (Table 1). *In vitro* studies show that such stimuli include hypoxia/reoxygenation [31,32,50,51,52], oxidants [33,53,54], UV light [38,39,55], heat stress [56], oxalate [54,57], P-fimbriae of *E. coli* [58,59], nephrotoxins, including cadmium [60,61], isoflurane [62], microcystin [63], nickel [64], and radiocontrast [65], Shiga-toxin B [58], staphylococcal enterotoxin B [66], interleukin (IL)-1β [59] and TNF-α [54,59]. *In vivo* studies show that ceramide is accumulated in kidneys exposed to anti-glomerular membrane (GBM) antibody [52], nephrotoxins such as carbon tetrachloride [67] and isoflurane [62], developing kidney [68,69], ischemia/reperfusion (I/R) injury [51,52], glycerol-induced myohemoglobinuria [52], and obstructive nephropathy [70]. These stimuli can induce apoptosis, and an apoptogenic role of ceramide is further supported by the ability of exogenous ceramide to induce apoptosis in RTCs [50,63,68,71].

**Table 1:** Ceramide/sphingosine generation in renal tubular cells after exposure to various stimuli.

Stimuli	Species	Cell Line for *in Vitro* */ Tissue for *in Vivo*	Increased Sphingolipid	Enzymes Involving Ceramide Metabolism	Ref.
*In vitro* study					
Cadmium	Rat	PTCs	Ceramide	CerSs↑	[60,61]
Heat stress	Dog	MDCK cells	Ceramide	CerSs↑	[56]
Hypoxia/reoxygenation	Pig/Rat	LLC-PK1 cells/NRK-52E cells	Ceramide	CerSs↑	[31,32,50]
	Mouse	PTCs	Ceramide	A-SMase↓, N-SMase↓	[51,52]
Interleukin-1β	Human	A498 cells **	Ceramide	A-SMase↑, N-SMase↑	[59]
Isoflurane	Mouse Human	PTCs/HK-2 cells	Ceramide/Sphingosine	A-SMase→, N-SMase→, CerS→	[62]
Microcystin	Human	HEK293 cells	Ceramide	unknown	[63]
Nickel	Rat	PTCs	Ceramide	CerSs↑	[64]
Oxalate	Pig/Dog	LLC-PK1 cells/MDCK cells	Ceramide	CerSs↑, SMases↑	[54,57]
Oxidants	Human	HK-2 cells	Ceramide/Sphingosine↓	N-CDases→, N-SMase↓, A-SMase→, CerSs→	[53]
	Pig/Dog	LLC-PK1 cells/MDCK cells	Ceramide	CerSs↑	[33,54]
P-finbriae of *E. coli*	Human	A498cells **	Ceramide	A-SMases→, N-SMase→, A-SMase↓, N-SMase→	[58,59]
Radiocontrast	Pig	LLC-PK1 cells	Ceramide	CerSs↑	[65]
Shiga-toxin B	Human	A498cells **	Ceramide	A-SMase→, N-SMase→	[58]
St. enterotoxin B	Human	PTCs	Ceramide	N-SMase↑	[66]
TNF-α	Dog/ Human	MDCK cells/A498 cells **	Ceramide	A-SMase↑, N-SMase↑	[54,59]
Ultraviolet light	Mouse	BMK cells	Ceramide	CerSs↑	[38]
	Human	HEK293 cells	Ceramide	CerS1 translocation from the ER to Golgi↑	[39]
	Human	HEK293 cells	Ceramide	A-SMase↑	[55]
*In vivo* study					
Anti-GBM Ab-induced ARF	Mouse	Kidney	Ceramide	A-SMase↑, N-SMase↑	[52]
Carbon tetrachloride	Rat	Kidney	Ceramide	A-SMase→, N-SMase↑	[67]
Developing kidney	Rat	Kidney	Ceramide/S1P↓	CerSs↑, SphKs↓	[68,69]
Ischemia/reperfusion	Mouse	Kidney	Ceramide	A-SMase↓, N-SMase↓	[51,52]
Isoflurane	Mouse	Kidney	Ceramide/Sphingosine	A-SMase→, ↑ ^#^, N-SMase→, CDase↑	[62]
Myohemoglobinuria	Mouse	Kidney	Ceramide	A-SMase↓, N-SMase↓	[52]
Ureteral obstruction	Rat	Kidney	Ceramide	unknown	[70]

* All types belong to the epithelial or tubular cell lines in the kidney; ** A498 cells: human kidney carcinoma cells; ^#^ Activity of A-SMase but not N-SMase was increased when the mouse normal kidney extracts were incubated with sphingomyelin in the presence of isoflurane; ARF, acute renal failure; A-SMase, acid sphingomyelinase; BMK, baby mouse kidney; CDase, ceramidase; CerSs, ceramide synthases; ER, endoplasmic reticulum; GBM Ab, glomerular basement membrane antibody; N-SMase, neutral sphingomyelinase; PTCs, proximal tubular cells; SMases, sphingomyelinases; SphKs, sphingosine kinases; S1P, sphingosine-1-phosphate; St., staphylococcal; TNF, tumor necrosis factor; ↑, increase; ↓, decrease; →, no change in the expression and activity.

*In vitro* studies show that SMases [54,57], including A-SMase [52,54,55,59], N-SMases [52,54,59,62,66], and CerSs [31,32,33,38,50,54,56,57,60,61,64,65,68,69] are involved in ceramide production in RTCs in response to cytokines, endotoxin, heat stress, hypoxia/reoxygenation, nephrotoxin, oxalate or oxidants. Shiga toxin, which can induce hemolytic uremic syndrome (HUS), increases ceramide formation [58], leading to apoptosis in RTCs [72]. This suggests that ceramide may regulate apoptosis in RTCs associated with HUS. *In vivo* studies show that ischemia/reperfusion (I/R) injury and glycerol-induced myohemoglobinuria are associated with reduced activity of both A-SMase and N-SMase despite an increase in ceramide levels in RTCs [52]. In contrast, the activity of SMases or CDase is increased in the kidneys exposed to anti-GBM antibody [52], carbon tetrachloride [67] or isoflurane [62]. Enhanced activity of CerSs, in association with reduced SphKs activity and S1P levels [69], are also found in the developing kidneys [68,69].

Ceramide accumulation and ceramide-dependent or ceramide-independent apoptosis vary with cell types and stimuli. For example, UV-irradiation induces ceramide production in HEK293 cells and Jurkat cells, whereas inhibition of ceramide production rescues UV-induced apoptosis in HEK293 cells but not in Jurkat cells [55]. Heat stress induces more production of ceramide in MDCK cells than in COS7 cells [56]. Oxidants induce ceramide generation in HK-2 cells [53] and LLC-PK1 cells [33] but not in HEK293 cells [73]. Ceramide exacerbates adenosine 5'-triphosphate (ATP) depletion/Ca^2+^ ionophore- or phospholipase A2 (PLA2)-induced cytotoxicity in HK-2 cells, whereas it attenuates arachidonic acid-induced cytotoxicity [51]. In addition, exogenous ceramide induces apoptosis in RTCs [50,63,68,71], but not in glomerular mesangial cells [74].

### 4.3. A Role of Balance between Ceramide and Sphingosine-1 Phosphate (S1P) in RTCs

#### 4.3.1. Sphingosine/Sphinganine

In the kidney field, Zager and his colleagues first described that high dose of sphingosine induces necrosis in HK-2 cells, whereas subtoxic dose renders the cells resistant to ATP depletion/Ca^2+^-ionophore-mediated injury [75]. Ischemia induces ATP depletion and a reduction of sphingosine and ceramide levels in the kidney, and sphingosine returns to normal levels and ceramide rises to supranormal levels during reperfusion, suggesting that sphingosine and ceramide fluxes can induce renal I/R injury [51]. Sphingosine and sphinganine induce apoptosis in RTCs [76], and these effects are potentiated by a SphK inhibitor and attenuated by S1P [76]. Fumonisin B1, an inhibitor of CerSs, which result in accumulation of sphinganine, can activate caspase-3 and apoptosis in human RTCs [77]. Inhibition of sphinganine using myriocin, a SPT inhibitor, protects fumonisin B1-induced cytotoxicity in LLC-PK1 cells [78]. These lines of evidence suggest that sphingosine/sphinganine mainly function as pro-apoptotic factors and that its biological effects may vary with the extent of their intracellular levels in RTCs.

#### 4.3.2. Ceramide and S1P

##### Sphingosine Kinases (SphKs)

Intracellular S1P levels are largely controlled by its formation via phosphorylation of sphingosine by SphKs, and to a lesser extent by degradation via S1P phosphatase localized in the ER (reversible) or S1P lyase (irreversible) [1,2]. There are two isoforms, SphK1 and SphK2, with different sequence, catalytic property, subcellular localization, and function. SphK1 functions as an anti-apoptotic factor, and is localized in the cytosol and translocated into the plasma membrane upon stimuli. SphK2 has a putative BH3-only motif, thereby functions as a pro-apoptotic factor through the mitochondrial pathway, and shuttles between the cytosol and nucleus upon stimuli [1]. Over-expression of SphK2 increases incorporation of palmitate into ceramide, whereas that of SphK1 decreases this process in HEK293 cells [47], indicating that SphK1 decreases ceramide formation, while SphK2 increases it in RTCs. Downregulation of SphK2 reduces conversion of sphingosine to ceramide in the salvage pathway, whereas downregulation of SphK1 enhances this process, resulting in increased ceramide levels [47], suggesting that SphK1 and SphK2 have the opposite effects on the maintenance of intracellular ceramide levels.

##### S1P/S1P Receptors as a Survival Factor

S1P exerts its biological effects as a survival factor through its binding to one or more of five S1P receptors (S1PRs) [S1P(1–5)R] on the cell surface, initiating G protein-coupled receptor signaling [1]. Glomerular mesangial cells express S1P(1–5)R [42,79], and RTCs have S1P(1–4)R but not S1P(5)R [37,42,44,45,79,80,81,82]. S1P(2)R is expressed mainly in RTCs and its expression is more abundant in collecting ducts and distal tubules than other segments [83].

I/R injury activates SphK1 but not SphK2 in the kidneys [84]. Isoflurane, an anesthetic agent, protects renal I/R injury through ERK-dependent SphK1 activation [85], leading to increased S1P levels [86]. Over-expression of SphK1 protects I/R-induced apoptosis of RTCs via enhanced expression of heat shock protein (HSP)-27, and functional protection and induction of HSP27 is blocked with S1P(1)R antagonism [84]. IL-11 [87] and activation of A(1) adenosine receptor, A(1)AR, can protect I/R-induced apoptosis of RTCs by enhancing SphK1 activity, resulting in accumulation of S1P and hypoxia-inducible factor (HIF)-1α that increases SphK1 activity [37]. In addition, increased nuclear HIF-1α is critical in mediating the renoprotective effects of S1P(2)R inhibition in renal I/R injury [82]. A selective S1P(1)R agonist up-regulates SphK1 activity and attenuates hypoxia/reoxygenation-induced apoptosis through activation of extracellular signal-regulated kinase (ERK) and/or phosphatidylinositol-3 kinase (PI3K)/Akt (known as protein kinase B) pathways in mouse RTCs [81]. In addition, a S1P(2)R antagonist up-regulates SphK1 and attenuates renal I/R injury through activation of SphK1/S1P(1)R signaling pathway, whereas a S1P(2)R agonist exacerbates the injury [82]. The effect of S1P(2)R antagonism takes place selectively in RTCs but not in renal endothelial cells [82]. Thus, a role of S1P/S1PRs-induced signaling pathway varies with cell types.

Over-expression of SphK1 protects apoptosis of RTCs in response to other stimuli. For example, it protects hydrogen peroxide-induced cell death of RTCs [80]. A selective S1P(1)R agonist up-regulates SphK1 and attenuates lipopolysaccharide (LPS)-induced apoptosis through activation of ERK and/or PI3K/Akt pathways in mouse RTCs [81]. A S1P(2)R antagonist also up-regulates SphK1 activity and attenuates hydrogen peroxide-induced necrosis and TNF-α/cycloheximide-induced apoptosis of RTCs through activation of SphK1/S1P(1)R signaling pathway, whereas a S1P(2)R agonist exacerbates the injury [82]. These data suggest that S1P(1)R and S1P(2)R-dependent signaling pathways differentially regulate ceramide-induced apoptosis of RTCs. This is further supported by the fact that inhibition of SphK1 exacerbates the injury, whereas a lack of SphK2 confers protection against renal I/R injury [84]. Inhibition of SphK1 also enhances ceramide accumulation and cadmium-induced apoptosis in MDCK cells [88], and exacerbates sphingonine- or sphinganine-induced apoptosis in HEK293 cells, whereas S1P by itself prevents this type of apoptosis [76]. A selective S1P(1/3)R antagonist and SphK inhibitors abrogate isoflurane-induced protection against hydrogen peroxide-induced necrosis through activation of ERK and Akt and induction of HSP70 [80]. These data suggest that SphK1/S1P(1,3)R signaling pathway, which enhances S1P accumulation, functions as anti-apoptotic, whereas SphK2/S1P(2)R signaling pathway serves a pro-apoptotic factor in ceramide-induced apoptosis of RTCs. Thus, modality for regulation of SphKs/S1PRs signaling pathways may have therapeutic potential for prevention of ceramide-induced kidney injury [81].

##### S1P Phosphatase and S1P Lyase as Pro-Apoptotic Factors

S1P phosphatase is abundant in the kidney; it degrades S1P and resides in the ER [89] (Figure 1). Over-expression of S1P phosphatase or S1P lyase results in accumulation of sphingosine and ceramide via degradation of S1P, thereby enhancing apoptosis of RTCs in response to TNF-α [89], oxidative stress [90], or stimuli including DNA damage [91]. In contrast, knockdown of S1P phosphatase increases S1P levels and renders HEK293 cells resistant to TNF-α-induced apoptosis [89]. In addition, SphK activity exceeds S1P phosphatase activity in embryonic kidneys, which results in a net accumulation of S1P, thereby reducing apoptosis [92]. These data suggest that S1P functions as a survival factor and that S1P phosphatase or S1P lyase function as pro-apoptotic factors. Thus, the balance between activity of SphK, S1P phosphatase and S1Plyase can regulate ceramide-induced apoptosis of RTCs [76].

### 4.4. Ceramide Compartmentalization and Trafficking in Ceramide-Induced Apoptosis

Compartmentalization and trafficking of ceramide play an important role in ceramide-induced apoptosis in various cells, including RTCs. Ceramide compartmentalization and trafficking were discussed in Section 3. Disassembly of the Golgi, together with inactivation of CERT via cleavage by caspases, can reduce ceramide trafficking and SM synthesis, thereby enhancing apoptotic cellular response [93]. Upon apoptotic stimuli, *de novo* generated ceramide in the ER can be transferred to mitochondria through the formation of MAMs, resulting in MOMP and subsequent initiation of apoptotic process [21]. Mitochondrial CerSs are activated by apoptotic stimuli [4]. Activity and subcellular localization of N-SMase-2 are regulated by oxidants, leading to its preferential trafficking from the Golgi to the plasma membrane, where it generates ceramide and initiates apoptotic process [94]. Apoptotic stimuli such as FasL can induce lysosomal trafficking and targeting of A-SMase, which forms lipid raft signaling platforms in the membrane of vascular endothelial cells, where ceramide is enriched by an action of lysosomal A-SMase that is translocated into the cell membrane through a direct fusion of lysosomes to the plasma membrane [95]. In addition, SphK1 is localized in the cytosol, but translocated into the plasma membrane upon apoptotic stimuli [1].

Little is known about a role of ceramide compartmentalization and trafficking in ceramide-induced apoptosis of RTCs. Albumin with fatty acid impurities or conjugated with palmitate but not albumin itself can increase mitochondrial ceramide synthesis, leading to apoptosis in RTCs [96]. Ceramide trafficking facilitates translocation of protein kinase C (PKC)-α to the Golgi apparatus in HEK293 cells [97], and this process may be involved in ceramide-induced apoptosis of RTCs [98]. Ceramide generated by endolysosomal hydrolysis of SM by an action of A-SMase activity can be endocytosed and converted to the apical ceramide-enriched compartment (ACEC), and ceramide associates with a cliogenic protein complex such as atypical PKC and Cdc42 at the ACEC in MDCK cells [25,26]. This novel function of ceramide for vesicular membrane transport and assembly of lipid-protein complex may regulate apoptotic signaling pathways in RTCs [99]. Thus, differential subcellular localization of the enzymes involved in ceramide metabolism and ceramide trafficking may play a crucial role in sphingolipids biosynthesis/turnover and apoptotic signal transduction in ceramide-induced apoptosis of RTCs.

## 5. Ceramide-Induced Signaling Pathway for Apoptosis

### 5.1. Overview of a Role of Mitochondria in Ceramide-Induced Apoptosis

**Figure 3:**
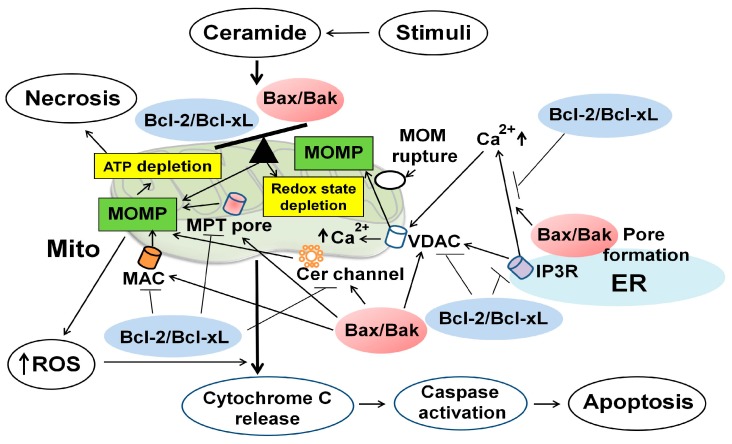
Mitochondria, Bcl-2 family proteins, and reactive oxygen species (ROS) in ceramide-induced apoptosis of renal tubular cells (RTCs). Once ceramide is synthesized and accumulated upon stimuli, it can induce ROS generation and depletion of redox system by inhibiting mitochondrial respiratory chain complexes. Depletion of ATP can switch the cell death from of apoptosis to necrosis. Ceramide enhances the expression and activity of pro-apoptotic Bcl-2 family proteins (e.g., Bax/Bak) and reduces those of anti-apoptotic Bcl-2 proteins (e.g., Bcl-2/Bcl-xL). Ceramide can regulate at least 4 channel/pore-forming complexes that regulate the MOMP: (1) a proteolipid pore, mitochondrial apoptosis-induced channel (MAC), formed by oligomerization of Bax and Bak mediated by tBid in the MOM; (2) a lipid channel formed by ceramide in the MOM; (3) a voltage-dependent anion channel (VDAC) in the MOM; and (4) MPT pore formed in the MIM in response to apoptotic stimuli. Ceramide can form MAC caused by oligomerization of Bax/Bak. Ceramide also assembles ceramide channel, which is regulated by Bcl-2 family proteins. Ceramide may form pores in the MOM, in concert with VDAC, which is not part of MPT pore, and possibly Bax and Bak. Additionally, ceramide may regulate the function of VDAC, which is regulated by several pro- and anti-apoptotic Bcl-2 family members. These processes lead to MOMP, thereby initiating apoptosis. BcL-2/Bcl-xL has the opposite function that inhibits MOMP. Ceramide can increase MPT pore opening, leading to MOMP. MOMP can enhance ROS generation and the release of apoptogenic proteins such as cytochrome C, which activate a caspase cascade, leading to apoptosis. The MOM is finally ruptured and this in turn contributes to mitochondrial remodeling, fusion and fission, which results in further release of apoptogenic factors, leading to apoptosis. There is an interconnection between the mitochondria and the ER through the regulation of calcium homeostasis via inositol 1,4,5-triphosphate receptor (IP3R) on the ER membrane. Bax/Bak can increase the release of Ca^2+^ from the ER into the cytosol via pore forming complexes on the ER membrane, whereas Bcl-2/Bcl-xL can prevent this event by directly or indirectly regulating IP3R. This process regulates mitochondrial calcium homeostasis, thereby regulating MOMP and apoptosis. ER, endoplasmic reticulum; Mito, mitochondria; ↑, increase; ┤, suppress the expression and activity.

Mitochondria have long been exclusively considered as “the powerhouse” of the cell, serving maximal energy production by oxidative phosphorylation. However, accumulating evidence indicates that mitochondria play a central role for apoptosis by regulating a variety of apoptotic signaling pathways. Mitochondria supply energy in the form of ATP, which is required for activation of caspase-9 [100]. Depletion of intracellular ATP can switch the cell death from apoptosis to necrosis [100] (Figure 3). Mitochondria produce ROS as a function of electron transport and redox status within the mitochondrial inner membrane (MIM) [101]. MOMP enhances ROS generation through the mitochondrial permeability transition (MPT) or by oxidized cytochrome C, which in turn activates a caspase cascade [101]. Once these cellular processes are initiated upon apoptotic stimuli, many intracellular signaling molecules are activated or inhibited, driving the terminal events of apoptosis.

Besides ATP synthesis and supply, mitochondria function as initiators and transducers of cell signaling by serving as the platforms for protein-protein signaling interactions and by regulating intracellular signaling molecules. In addition, mitochondria emerge as an important intracellular compartment of sphingolipids metabolism because the mitochondrial enzymes involved in sphingolipids metabolism can regulate ceramide formation. Ceramide can induce apoptosis specifically when generated in mitochondria [4], and in turn mitochondria play a central role for the regulation of ceramide-induced apoptosis. Ceramide has various effects on mitochondria, including ATP depletion, alteration of mitochondrial Ca^2+^ homeostasis, collapse in the mitochondrial membrane potential (Δψm), inhibition of the mitochondrial electron transport chain complex, enhanced ROS generation [102], and the release of intermembrane space apoptogenic proteins [4]. In this section, I will review the current evidence for a role of mitochondria in ceramide-induced apoptosis in various cells, including of RTCs.

### 5.2. Mitochondrial Outer Membrane Permeability (MOMP) and Bcl-2 Family Proteins

**Figure 4:**
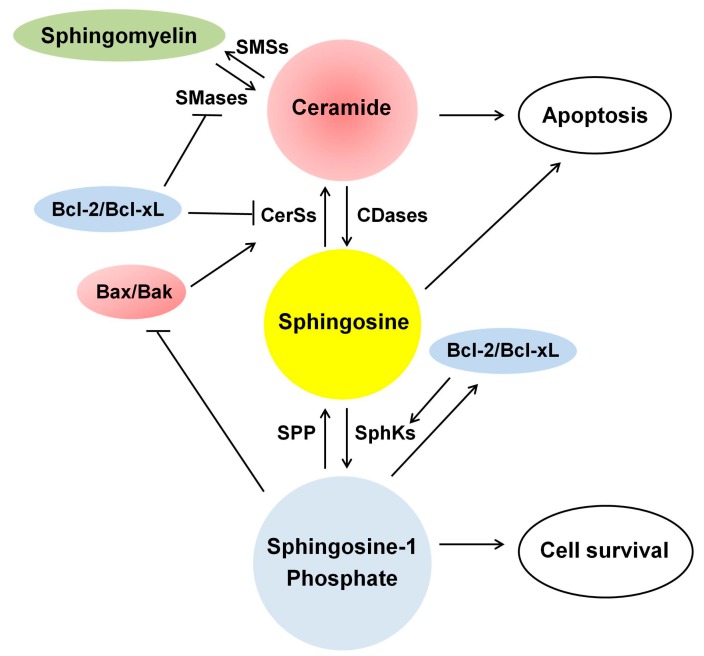
Ceramide, sphingosine and sphingosine-1-phosphate (S1P) and their regulation by Bcl-2 family proteins in apoptosis. Ceramide and sphingosine function as pro-apoptotic factors, while S1P functions as a survival factor. Bcl-2/Bcl-xL activates sphingosine kinases (SphKs), resulting in accumulation of S1P, thereby reducing apoptosis. Bcl-2/Bcl-xL suppresses CerSs and SMases, reducing ceramide accumulation and apoptosis. In contrast, Bax/Bak increases CerSs activity, thereby enhancing ceramide accumulation and apoptosis. S1P prevents apoptosis by reducing the expression and activity of Bax/Bak and activating those of Bcl-2/Bcl-xL. S1P lyase degrades S1P, reducing S1P levels, thereby enhancing apoptosis. CDases, ceramidases; CerSs, ceramide synthases; SMSs, sphingomyelin synthases; SPP, S1P phosphatase; ↑, increase; ┤, suppress the expression and activity.

Ceramide can be generated not only in mitochondria but also in microsomes of the ER and the plasma membrane that can be transferred to mitochondria [21,22], resulting in increased mitochondrial ceramide levels. Ceramide also reduces the expression of Bcl-2 and Bcl-xL proteins that can inhibit N-SMase activity [103] (Figure 3 and Figure 4). Thus, mitochondria can contain high concentration of ceramide in the MOM upon apoptotic stimuli.

Increased mitochondrial ceramide levels can in turn induce MOMP, resulting in ATP depletion, perturbation of Ca^2+^ homeostasis, dimerization of Bcl-2 family proteins in the MOM, ROS generation, collapse in the MIM potential, inhibition and/or activation of various components of the mitochondrial electron transport chain complex, and release of intermembrane space proteins, leading to MOMP and initiation of apoptosis [4]. Mitochondria play a central role in the regulation of ceramide-induced apoptosis through MOMP [4,5], The loss of integrity of MOM, leading to MOMP, is considered the point of no return for apoptotic process since the cell commit to die once MOMP is initiated. Currently, the mechanism by which ceramide induces MOMP is not precisely known and is a matter of debate.

#### 5.2.1. Mitochondrial Integrity Regulated by Bcl-2 Family Proteins

The Bcl-2 family proteins are divided into three groups based on the Bcl-2 homology (BH) domains [49,104]. Anti-apoptotic multidomain proteins (Bcl-2, Bcl-xL, BcL-W, Mcl-1 and A1) contain four BH domains (BH1–4). They are generally integrated within the MOM, but may also be in the cytosol or ER membrane. Upon stimuli, they associate with or integrate mainly into the MOM. The anti-apoptotic Bcl-2 family proteins preserve the MOM integrity by directly inhibiting the pro-apoptotic Bcl-2 family proteins.

Pro-apoptotic Bcl-2 family proteins are divided into the effector proteins and the BH-3 only proteins. The effectors such as Bax and Bak contain three BH domains (BH1–3). Bax and pro-apoptotic BH3-only proteins are predominantly localized in the cytosol, while Bak (pro-apoptotic) is intrinsic MOM protein as Bcl-2. Upon apoptotic stimuli, Bax and pro-apoptotic BH3-only proteins are translocated from the cytosol to the MOM, while Bak is activated and oligomerized in the MOM. Pro-apoptotic BH3-only proteins are subdivided based on their ability to interact with the anti-apoptotic Bcl-2 proteins or both the anti-apoptotic proteins and the effectors. BH3-only proteins that only bind to the anti-apoptotic Bcl-2 family members are termed “sensitizer” and/or “derepressor” BH-3 only proteins (Bad, Bik, BMF, HRK, Noxa and Puma). The BH3-only proteins (e.g., Bid and Bim) that interact with both the anti-apoptotic Bcl-2 family members and the effectors can induce oligomerization of Bax and Bak in the MOM in several ways, leading to MOMP [49,104]. In direct activation model, BH3-only proteins as activator proteins (e.g., Bid, Bim, Puma) bind to and activate Bax, thereby leading to integration and oligomerization of Bax in the MOM [104]. In addition, one activated Bax may recruit additional Bax. On the other hand, Bcl-xL sequesters the BH-3-only activator proteins, thereby prevents Bax activation, leading to inhibition of MOMP. In the displacement model, anti-apoptotic Bcl-2 family proteins bind to and thereby inhibit active forms of Bax/Bak. The BH3 only proteins displace Bax and Bak from anti-apoptotic proteins (Bcl-xL, Mcl-1 and Bcl-w). The released Bax then inserts into the MOM and oligomerizes in the MOM, leading to MOMP. In the embedding together model, Bax binds to MOM peripherally concomitant with a conformational change in Bax that increases the affinity and interaction of Bax and truncated (t)Bid cleaved by caspase-8. The interaction between MOM bound tBid with peripherally membrane bound Bax triggers insertion of Bax into the MOM. The integrated Bax recruits additional Bax and oligomerizes, leading to the MOMP. Bak can function similarly to Bax except that it binds to the different modulators (e.g., tBid, Bim). In this way, MOMP is tightly regulated by the Bcl-2 family proteins; pro-apoptotic (MOMP-promoting) and anti-apoptotic (MOMP-inhibiting) proteins.

#### 5.2.2. Ceramide-Induced Bax/Bak Pore Formation in Mitochondria

Ceramide is implicated at least in four different channel/pore-forming complexes at mitochondria (Figure 3 and Table 2): (1) a proteolipid pore formed by oligomerization of Bax/Bak via tBid in the MOM, termed mitochondrial apoptosis-induced channel (MAC); (2) ceramide channel in the MOM; (3) a voltage-dependent anion channel (VDAC) in the MOM; and (4) a large conductance pore-forming complex, termed mitochondrial permeability transition (MPT) pore that is formed in the MIM. Ceramide can enhance mitochondrial translocation of pro-apoptotic Bcl-2 family protein, Bax, from the cytosol to the MOM, while pro-apoptotic Bak is predominantly localized in the MOM and thus Bak is activated and oligomerized in the MOM [49,104]. Oligomerization of Bax/Bak forms MAC in the MOM, and this process is tightly regulated by anti-apoptotic Bcl-2 family proteins [105,106,107], Ceramide reduces cellular expression of anti-apoptotic Bcl-2 proteins (e.g., Bcl-2/Bcl-xL), thereby further enhancing formation of MAC in the MOM. These processes can enhance MOMP, which results in not only enhanced ROS generation, but also the release of cytochrome C into the cytosol and caspase activation, leading to apoptosis.

The enzymes involved in ceramide metabolism can regulate MOMP by affecting Bcl-2 family proteins. Using isolated mitochondria from murine liver, N-SMase, in cooperation with tBid, induces oligomerization and conformational change of Bax, leading to MOMP, whereas Bcl-xL and N-SMase inhibitor can block both Bax- and Bak-dependent MOMP [108] (Table 2). In addition, Bak/Bax-dependent MOMP requires different components of the sphingolipids pathway; Bak cooperates with S1P, whereas Bax cooperates with hexadecenal [108]. A-SMase-dependent ceramide generation induces Bax conformational change and down-regulation of anti-apoptotic Bcl-2 and Bcl-xL proteins, leading to MOMP, while Bcl-2 inhibits MOMP and A-SMase activation is indispensable for Bax change [109]. Upon irradiation, mitochondrial ceramide-rich macrodomain (MCRP) coupled to CerS-mediated ceramide generation can optimize Bax insertion/oligomerization in the MOM, leading to MOMP [110]. In these studies, MAC is likely to be responsible for MOMP although a role of Bax/ceramide channel, which may be different from that formed by Bax alone or ceramide alone (ceramide channel) [5], cannot be excluded.

In RTCs, radiocontrast reduces Bcl-2 and increases Bax, which is reversed by inhibition of CerS, and C2-Cer produces similar changes [65] (Table 2). Ceramide can induce apoptosis through mitochondrial translocation of Bax from the cytosol, altering MOMP, thereby resulting in the release of cytochrome C and caspase activation in RTCs [71]. In fact, glioma cells overexpressing Bax undergo apoptosis in response to ceramide in caspases-dependent manner [103], and HL-60 cells with reduced expression of Bax are resistant to ceramide-induced apoptosis [111]. These data suggest a mutual relationship between ceramide metabolism and Bcl-2 family proteins in the regulation of MAC, leading to MOMP.

#### 5.2.3. Ceramide Channel Regulated by Bcl-2 Family Proteins

Ceramide with various chain lengths (C2 and C16) at physiological concentration forms pores in phospholipid membranes, composed of a ring of anti-parallel columns with six ceramide molecules linked by hydrogen bonds [112,113]. Ceramide forms channel in planar membranes, lysosomes, microsomes, and mitochondrial membranes [5,112]. The formation of ceramide channel in the MOM is directly correlated with ceramide concentration [113,114], which is modulated by activated but not monomeric Bax [112] (Table 2). Thus, accumulated ceramide in mitochondria, in conjunction with activated Bax, can promote the formation of ceramide channels in the MOM and subsequently increase MOMP, resulting in the release of pro-apoptotic proteins within the mitochondrial intermembrane space and initiation of apoptosis.

Although Bcl-2 family proteins are not required to form ceramide channel in the MOM [114], the formation of ceramide channel is tightly regulated by Bcl-2 family proteins [5,112,114,115]. Pro-apoptotic Bax directly enhances the formation of ceramide channel [112] through its ability to interact with ceramide channel via the configuration of C2-hydroxyl group of ceramide [115]. Bax acts synergistically with ceramide to enhance MOMP through their direct interaction to form ceramide channel without requirement of Bid or Bak [112]. Activated Bax favors the growth of ceramide channel and increases the size of the single existing ceramide channel [112]. On the other hand, anti-apoptotic Bcl-2, Bcl-xL and CED-9 antagonize the formation of ceramide channel and disassemble ceramide channels in the MOM through direct interaction with anti-apoptotic Bcl-2 family members and ceramide channels [112,114]. In addition, Bcl-xL inhibits ceramide channel formation through interaction of its hydrophobic groove with the ceramide molecules [115].

As another regulatory mechanism of ceramide channel formation, low dose of sphingosine potentiates channel formation by long-chain ceramide but not by short-chain ceramide, whereas high concentration of sphingosine may intercalate into the ceramide channels, resulting in their destabilization because sphingosine lacks the amide linkage and has a net charge that leads to electrostatic repulsion [116]. Dihydroceramide, which is converted to ceramide by dihydroceramide desaturase, can also inhibit ceramide channel formation in the mitochondria [117].

#### 5.2.4. Voltage-Dependent Anion Channel (VDAC) and Mitochondrial Permeability Transition (MPT) Pore

It has been controversial whether or not VDAC is part of MPT pore. However, a recent genetic study using isolated mitochondria from the cells lacking VDACs has reconciled that VDAC is dispensable for MPT pore function, suggesting that VDAC is not part of MPT pore [118]. The VDAC has three isoforms (VDAC1–3) and is the most abundant integral membrane protein of the MOM where it forms hydrophilic pores [118]. Over-expression of VDAC renders the cells more susceptible to ceramide-induced apoptosis since VDAC enhances the amplitude of the agonist-dependent increase of mitochondrial matrix Ca^2+^ concentration by allowing the fast diffusion of Ca^2+^ from the ER release sites to the MIM [119]. Ceramide may also form channels in the MOM, in concert with VDAC and possibly Bax, that allow the release of pro-apoptotic factors from mitochondria, leading to apoptosis [120]. Additionally, an inhibitor of VDAC rescues ceramide-induced apoptosis in human fibroblasts, suggesting that ceramide may regulate the function of VDAC [120]. Several Bcl-2 family members interact with VDAC and regulate apoptosis through induction of either opening or closure of VDAC [121]. Bax is preferentially associated with mitochondrial detergent-resistant membranes (mDRMs), which contain abundant ceramide and cholesterol and associate with the VDAC and adenine nucleotide translocase (ANT), a specific ATP/ADP transporter [122]. These data suggest that membrane microenvironments enriched in ceramide and cholesterol in mitochondria favor interaction between VDAC and Bax in the MOM, leading to MOMP.

Ceramide induces protein phosphatase A2 (PPA2)-dependent dephosphorylation of the BH3-only protein, Bad, which sensitize MPT pore to Ca^2+^ through interaction with Bad, Bcl-xL, and VDAC without requirement of Bax or Bak in HEK293 cells, although the role of VDAC in ceramide-induced dephosphorylation of Bad for sensitization of MPT pore is rather indirect [123] (Table 2). Positively charged ceramide accumulated in the mitochondrial matrix can increase MPT pore permeability, leading to MOMP [124]. Taken together, ceramide affects VDAC and MPT pore through interaction with Bcl-2 family proteins, leading to apoptotic process in RTCs.

**Table 2:** Regulation of ceramide-induced mitochondrial outer membrane permeability (MOMP) by Bcl-2 family proteins.

Stimuli	Cell Line/Tissue	Enzymes Involving MOMP	Bcl-2 Proteins that Regulate Cer-Induced MOMP	Channel for Cer-Induced MOMP	Comments	Ref.
In other types of cells						
t-Bid, N-SMase	Murine liver mo./HeLa cells	N-SMase↑	tBid-induced Bax oligomerization/ conformational change	MAC	Bax cooperates with Hex and Bak cooperates with S1P to induce t-Bid-mediated MOMP. Bcl-xL and N-SMase inhibitor inhibit MOMP.	[108]
UV light	HeLa cells	A-SMase↑	Bax conformational change	MAC	Bcl-2 inhibits MOMP. Bax change requires A-SMase activation.	[109]
C16-Cer	HeLa cells	NA	Bax conformational change	MAC	Cer but not UV induces Bax change in A-SMase-deficient cells. Bcl-2 prevents Cer-induced Bax change.	[109]
Irradiation	HeLa cells	CerS↑ in MAM	Bax↑	MAC	Oligomeric Bax insertion into MOM causes MOMP.	[110]
C16-Cer	Mo. of HeLa cells/ mouse liver	NA	tBid-induced Bax↑	MAC	Cer induces MCRM *, favoring Bax insertion to MOM and oligomerization.	[110]
C16-Cer	Rat liver mo.	NA	t-Bid-induced Bak↑	Cer channel	Cer and Bax synergistically induce MOMP. Oligomeric Bax enhances Cer channel formation.	[112]
C16-Cer	Rat liver mo.	NA	Bcl-xL/CED-9 prevents MOMP	Cer channel	Bcl-xL/CED-9 prevents and disassembles Cer channel.	[114]
In renal tubular cells						
Radiocontrast	LLC-PK1 cells	CerS↑	Bax↑, Bcl-2↓	probably MAC	CerS inhibition reverses the change in Bax/Bcl-2.	[65]
C2-Cer	HK-2 cells	NA	Bax↑	probably MAC		[71]
C16-Cer	BMK cells	NA	MOMP occurs in Bax^−/−^Bak^−/−^ cells	Cer channel	Bax/Bak is dispensable for Cer channel formation.	[114]
C2-Cer	HEK293 cells	NA	Bad dephosphorylation	MPT pore	Bad/Bcl-xL/VDAC but not Bax/Bak regulate MPT pore opening.	[123]

* MCRM consists of Cer, Bax, Bak, and VDAC; A-SMase, acid sphingomyelinase; BMK, baby mouse kidney; Cer, ceramide; CerS, ceramide synthase; Hex, hexadecenal; MAC, mitochondrial apoptosis-induced channel; MAM, mitochondrial-associated membrane; MCRM, mitochondrial ceramide-rich macrodomain; mo, mitochondria; MOMP, mitochondrial outer membrane permeability; N-SMase, neutral sphingomyelinase; S1P, sphingosine-1-phosphate; tBid, truncated Bid; UV, ultraviolet; VDAC, voltage-dependent anion channel; ↑, increase; ↓, decrease; NA, not applicable.

#### 5.2.5. Ceramide-Induced Mitochondrial Calcium Uptake and Fission Regulate MOMP

Ceramide induces excessive accumulation of Ca^2+^ in mitochondrial matrix that triggers opening of the MPT pore and MOMP in RTCs [123] (Table 2). As discussed later, ceramide can increase mitochondrial Ca^2+^ uptake from the release of Ca^2+^ in the ER via caspase 8-cleaved tBid [125], and over-expression of Bcl-2 prevents this event [126]. On the other hand, ceramide can induce mitochondrial fission via an increase in the expression of dynamin-related protein-1 (Drp1) and mitochondrial fission-1 protein (Fis1) [127], leading to MOMP. Bax/Bak can also induce mitochondrial fission [128], causing MOMP. Bak may collaborate with Bax to permeabilize the MOM by regulating mitochondrial fusion, and Bcl-2 and Bcl-xL inhibit mitochondrial fragmentation in baby mouse kidney (BMK) cells [129] although the ability of Bcl-2/Bcl-xL to prevent Bax/Bak-induced mitochondrial fission is controversial [130]. These data suggest that ceramide-induced mitochondria Ca^2+^ uptake and mitochondrial fission, which may be regulated by Bcl-2 family proteins, can regulate MOMP, leading to apoptosis.

#### 5.2.6. Ceramide-Induced MOMP in the Regulation of Apoptosis of RTCs

Little is known about a role of MOMP in the regulation of ceramide-induced apoptosis of RTCs. Ceramide can induce MOMP in Bax^−/−^Bak^−/−^ BMK cells, suggesting that Bax and Bak are dispensable for ceramide-induced MOMP in RTCs [114] (Table 2). In contrast, Bak/Bax is required for ceramide generation in UV irradiation-induced apoptosis of BMK cells [38]. Ceramide increases the mitochondrial translocation of Bax and activation of caspases, a downstream event of Bax, in the regulation of ceramide-induced apoptosis of RTCs [71]. Inhibition of CerSs but not SMases ameliorates radiocontrast media-induced MOMP, caspase-3 activation and apoptosis, and down-regulates the expression of Bcl-2 in LLC-PK1 cells [65]. Exogenous C2-ceramide causes similar events [65]. Exposure of MDCK cells to oxalate, which increases ceramide generation [131], can reduce Δψm, and exogenous C2-ceramide decreases Δψm, thereby inducing caspase activation and apoptosis in MDCK cells [57]. The Bcl-2 protein can prevent oxalate-induced apoptosis in RTCs [131]. Ischemia results in ceramide generation in RTCs [31,32], and ischemia-induced depletion of mitochondrial guanosine triphosphate (GTP) can induce apoptosis in RTCs [132]. In addition, repletion of GTP can prevent ischemia-induced apoptosis, suggesting a role of mitochondria in this model of injury [132]. On the other hand, ceramide can increase intracellular Ca^2+^ levels by enhancing activity of Ca^2+^-ATPase located at the basolateral membranes of RTCs [133], and this process might induce mitochondrial Ca^2+^ uptake, leading to MOMP and apoptosis in RTCs. During ATP depletion by hypoxia, which increases ceramide [31,32,50,52], Drp1 is dephosphorylated, thereby contributing to mitochondrial fission and MOMP, and leading to the release of apoptogenic factors and apoptosis in RTCs [134]. This finding suggests a role of mitochondria fusion in hypoxia-induced apoptosis in RTCs. Taken together, these lines of evidence suggest a role of ceramide-induced MOMP in the regulation of apoptosis of RTCs.

#### 5.2.7. Ceramide-Induced and Ceramide-Independent MOMP in Apoptosis

In cell free model, Bax by itself can form pores in pure liposomes that can trigger the release of cytochrome C [135]. Oligomeric Bax can form channels in lipid bilayers, whereas monomeric Bax has no such activity [136]. In addition, in the presence of tBid, Bax can form ionic channel in liposomes and planar bilayers, and this channel-forming activity is mediated by an interaction between tBid and Bax, and is inhibited by Bcl-xL [137]. These data suggest that ceramide-independent MOMP mediated by Bcl-2-family proteins may occur within the cell. However, mammalian cells contain ceramide not only as a structural component of membranes but also a “hub” of the complicated sphingolipids metabolism. Thus, it is impossible to knockout the genes for all enzymes involved in ceramide metabolism, and if possible, such knockout cells cannot survive. This makes it extremely difficult to discriminate ceramide-dependent MOMP from ceramide-independent MOMP within the cells under normal condition and those exposed to apoptotic stimuli.

So far, there has been no study showing the difference between ceramide-induced and ceramide-independent MOMP during the apoptotic process in mammalian cells. Palmitate can induce apoptosis in ceramide-independent pathway [138,139]. In addition, palmitate can induce ROS production and hydrogen peroxide release as well as a loss of mitochondrial potential, leading to apoptosis [140]. However, it remains unknown whether or not there is a difference between ceramide-dependent and ceramide-independent MOMP in terms of a role for the apoptotic process in mammalian cells.

### 5.3. Regulation of the Enzyme Involved in Ceramide Metabolism by Bcl-2 Family Proteins

Extensive studies have focused on a role of Bcl-2 family proteins in the regulation of mitochondrial function during apoptosis. However, recent studies point out an additional role of Bcl-2 family proteins in the regulation of the enzymes involved in ceramide metabolism (Figure 4). For example, Bak but not Bax is required for ceramide generation via activation of CerSs in a MOMP-independent manner in BMK cells [38]. Bak with tBid can increase CerSs activity in microsomes, while anti-apoptotic Bcl-2 protein, Mcl-1, inhibits CerSs activity in human leukemia cells [141]. Over-expression of Bcl-xL inhibits ceramide formation by repressing N-SMase, whereas Bax has no effect in glioma cells [103]. Bcl-2 reduces ceramide generation in human adenocarcinoma cells although the responsible enzyme for ceramide generation was not identified [142]. Over-expression of Bcl-2 also stimulates the expression and activity of SphK1, thereby decreasing ceramide/S1P ratio, leading to resistance to ceramide-induced apoptosis [143]. These data suggest a potential role of Bcl-2 family proteins in the regulation of the enzymes involved in ceramide metabolism.

### 5.4. Ceramide-Induced Generation of Reactive Oxygen Species (ROS) and Its Regulation by Bcl-2 Family Proteins

#### 5.4.1. Mitochondria and ROS Generation in Ceramide-Induced Apoptosis

Mitochondria are the most prominent source of intracellular ROS generation. Ceramide directly inhibits mitochondrial respiratory chain (complex I–IV) [102,144], resulting in ROS generation. In fact, mitochondrial respiration-deficient cells do not produce ROS in response to ceramide [102]. Ceramide not only enhances ROS production [102,144,145] but also decreases antioxidant redox system in various cells [145] (Figure 3).

Regarding a role of ROS in ceramide-induced apoptosis of RTCs, isolated renal mitochondria in response to oxalate and ceramide can enhance ROS generation, lipid peroxides, and deduced thiol proteins [57]. Increased ceramide generation via activation of N-SMase but not of A-SMase with concomitant decrease in antioxidants such as vitamins C and E and the ratio of reduced glutathione (GSH)/oxidized GSH (GSSG) occur in the kidneys exposed to carbon tetrachloride [67]. These data suggest that the excess accumulation of ceramide and ROS with decreased antioxidant system causes cytotoxicity in the kidney (Table 3). Exposure of glomerular mesangial cells to nitric oxide donors or superoxide-generating substances enhances ceramide formation and apoptosis [146,147]. Cadmium can increase ceramide formation, which occurs downstream of ROS formation in a cell line derived from the S1 segment of rat RTCs [61]. These data suggest that ceramide is either upstream or downstream event of ROS in ceramide-induced apoptosis, depending on cell types and stimuli.

#### 5.4.2. Can ROS and Redox State Regulate the Enzymes Involved in Ceramide Metabolism?

ROS can enhance activities of CerSs [33], A-SMase [148], and N-SMase2 [149,150] in various cells, resulting in ceramide production. ROS-induced activation of N-SMase is inhibited by an antioxidant GSH [150]. On the other hand, ceramide-induced ROS production can inhibit SphK1 activity [151], resulting in increased ceramide/S1P ratio, which in turn enhances apoptosis. Inhibition of SphK1 can enhance generation of ROS and ceramide, thereby increasing apoptosis [152]. Low concentration of ROS activates SphK1, but high concentration inhibits SphK1 [149], suggesting that the extent of intracellular ROS levels may regulate the enzymes involving ceramide metabolism although the mechanism remains elusive.

Redox status can regulate the enzymes involved in ceramide metabolism. GSH depletion can inhibit SMSs [153]. In addition, hydrogen peroxide can activate N-SMase2 but GHS abolishes N-SMase2 activity [154]. Subcellular localization of N-SMase2 can be controlled by oxidant stress, leading to its trafficking to the plasma membrane, where it generates ceramide and induces apoptosis [150]. UV irradiation can induce redox-dependent activation and relocalization of A-SMase to the external surface of raft membrane microdomains, resulting in SM hydrolysis in the plasma membrane outer leaflet, ceramide generation and apoptosis [155]. Over-expression of PKCζ abrogates these events and increases intracellular levels of antioxidant enzymes, thereby inhibiting A-SMase translocation [155]. Depletion of intracellular GSH can induce caspase-dependent inhibition of SMSs activity, ceramide generation, and apoptosis induced by curcumin [153]. Intracellular S1P can inhibit generation of ceramide and ROS, thereby reducing apoptosis in neuronal cells [152], and over-expression of SphK1 can reduce ceramide accumulation in skeletal muscle cells [156].

In the kidney, ROS can activate CerSs [31,32,33,54,57,65] and N-SMase2 [67] in RTCs after exposure to various stimuli (Table 3). An antioxidant, *N*-acetylcysteine, can prevent oxalate-induced ceramide accumulation by inhibiting CerSs or SMase in MDCK cells and LLC-PK1 cells [54]. In contrast, oxidant can suppress activity of N-SMase but not A-SMase in HK-2 cells [53]. Nitric oxide enhances N-SMase activity, resulting in increased ceramide levels in glomerular mesangial cells [147], and degradation of N-CDase through activation of the ubiquitin/proteasome complex [157], leading to apoptosis. ROS can activate SphK1 in glomerular mesangial cells [158]. In addition, *in vitro* GSH can activate N-SMase activity in HK-2 cells [53]. These data suggest that ROS and redox state can regulate the enzymes involved in ceramide metabolism, which varies with cell types, thereby differentially regulating apoptosis in the kidney.

#### 5.4.3. A Role of Bcl-2 Family Proteins and ROS Production in Ceramide-Induced Apoptosis

Anti-apoptotic Bcl-2 allows the cells to adapt to increased oxidative stress by enhancing intracellular antioxidant defense system, which counteracts ROS production in response to ceramide [159]. Bcl-2 can maintain the redox defense system or prevent ROS generation, thereby preventing ceramide-induced apoptosis [160]. Up-regulation of Bcl-xL can also prevent ceramide-induced ROS generation and apoptosis [161]. In contrast, pro-apoptotic Bax can increase ROS production in ceramide-induced apoptosis [162], and the cells lacking Bax do not produce ROS [163]. Bax inhibitor-1 (BI-1) can reduce ROS production during apoptosis [164], suggesting that Bax is upstream event of ROS formation. On the other hand, ROS can activate Bax [165], suggesting that Bax is downstream of ROS in ceramide-induced apoptosis. Thus, it depends on cell types whether Bcl-2 family proteins regulate ceramide-induced ROS generation or are downstream events of ROS, thereby regulating apoptosis.

In the kidney, radiocontrast media induces generation of ROS and ceramide in RTCs [166] (Table 3). The expression of Bax is increased and that of Bcl-2 is decreased in this model of injury [65]. Isolated mitochondria from rat kidneys in response to ceramide can induce ROS generation [57], and ceramide can induce mitochondrial Bax translocation in RTCs [71]. These data suggest that interaction between ROS and Bcl-2 family proteins may play a role in the regulation of ceramide-induced apoptosis of RTCs.

**Table 3:** Ceramide-induced ROS generation and ROS-induced regulation of the enzymes involving ceramide production and Bcl-2 family proteins in RTCs injury.

Stimuli	Cell Line/Tissue	Cer-Induced Alteration of ROS/AOS	Enzymes for Cer Production Regulated by ROS	Bcl-2 Proteins Regulated by ROS/AOS	Cell Death	Ref.
Hypoxia	LLC-PK1 cells, NRK-52E cells	ROS↑	CerS↑, A-SMase→, N-SMase→	unknown	Necrosis/Apoptosis	[31,32]
Oxidant	LLC-PK1 cells	Oxidant induces Cer	CerS↑, A-SMase→, N-SMase→	unknown	Apoptosis/Necrosis	[33]
Oxidant	HK-2 cells	Oxidant induces Cer	N-SMase↓, A-SMase→, CerS→, GSH activates N-SMase.	unknown	Necrosis	[53]
Oxalate	MDCK cells, LLC-PK1 cells	ROS↑	CerS↑, SMase↑	unknown	Apoptosis	[54,57]
Cadmium	Rat RTCs	ROS↑	unknown	unknown	Apoptosis	[61]
Carbon tetrachloride	Rat kidney	ROS↑, AOS↓	N-SMase↑, A-SMase→	unknown	Apoptosis	[67]
C2-Cer	HK-2 cells	ROS↑	NA	Bax↑	Apoptosis	[71]
Radiocontrast	LLC-PK1 cells	ROS↑	CerS↑	Bax↑, Bcl-2↓	Apoptosis	[65,166]

AOS, anti-oxidant system; A-SMase, acid sphingomyelinase; Cer, ceramide; CerS, ceramide synthase; GSH, glutathione; N-SMase, neutral sphingomyelinase; ROS, reactive oxygen species; RTCs, renal tubular cells; ↑, increase; ↓, decrease; →, no change in the expression and activity; NA, not applicable.

#### 5.4.4. Do Ceramide-Induced ROS Regulate the Expression of Bcl-2 Family Proteins in Ceramide-Induced Apoptosis?

Ceramide reduces the expression and activity of anti-apoptotic protein such as Bcl-2 and Bcl-xL and increases those of pro-apoptotic Bax and Bak, thereby leading to apoptosis in various cells [167,168] (Figure 3). Ceramide-induced ROS production results in more than 9-fold increase in the ratio of Bax/Bcl2 in breast cancer cells [168]. Radiocontrast, which enhances generation of ceramide and ROS, can increase Bax expression and decrease Bcl-2 expression in RTCs [65] (Table 3). Ceramide, which induces ROS production, can induce mitochondrial translocation of Bax in RTCs [71]. The Bcl-2 family proteins are targeted by ROS in cadmium-induced apoptosis of RTCs [169]. Ceramide-induced ROS acts as apoptotic signaling intermediate, leading to conformational activation of Bak/Bax, MOMP and apoptosis in leukemia cells [170]. These data suggest that ceramide-induced ROS generation may regulate the expression of Bcl-2 family proteins, thereby regulating ceramide-induced apoptosis of RTCs.

#### 5.4.5. A Role of Ceramide-Induced ROS in Apoptosis of RTCs

Oxalate and ceramide can increase the accumulation of ROS, lipid peroxides, and oxidized thiol proteins in RTCs, and redox state can inhibit oxalate-induced apoptosis [57] (Table 3). Hypoxia causes ceramide generation and ROS production in RTC injury [31,32]. Hydrogen peroxide can increase ceramide generation via activation of CerS but not A-SMase or N-SMase in microsomes, resulting in apoptosis in LLC-PK1 cells, suggesting that ROS is a regulator of CerSs and that ROS-induced ceramide generation plays a key role in the regulation of oxidant-induced RTC injury [33]. Carbon tetrachloride intoxication causes redox imbalance and oxidant stress, which in turn activates N-SMase but not A-SMase, leading to kidney injury [67]. Cadmium increases generation of ROS and subsequent ceramide generation and apoptosis in RTCs [61]. These data suggest that ceramide-induced ROS may affect a variety of downstream molecular events in ceramide-induced apoptosis. In the next section, I will discuss a role of ceramide-regulated molecular events in the regulation of apoptosis.

## 6. Interconnection between Mitochondria and ER in the Regulation of Calcium Homeostasis in Ceramide-Induced Apoptosis

### 6.1. Calcium Homeostasis in the ER and Ceramide-Induced Apoptosis

The ER and mitochondria are the major Ca^2+^ storage sites, and the fluxes of Ca^2+^ from the ER to mitochondria can regulate apoptosis (Figure 3). High Ca^2+^ concentration in the ER is transported to mitochondria, leading to MOMP. Ceramide increases intracellular Ca^2+^ through the release of Ca^2+^ from a Ca^2+^ channel, the inositol 1,4,5-trisphosphate receptor (IP3R) located in the ER [171]. Ca^2+^ is released primarily from the ER through IP3R, while Ca^2+^ reuptake is dependent on the sarcoplasmic-endoplasmic reticulum Ca^2+^-ATPase (SERCA) [172]. The ER and mitochondria are interconnected both physically and physiologically, and the physical linkage of the ER-mitochondria interface facilitates the exchange of Ca^2+^ and lipids between these two organelles. Upon stimuli, this process aligns these organelles tighter, and apoptotic cell signaling, including Ca^2+^, is relayed to the mitochondria, thereby initiating apoptosis. Once Ca^2+^ reaches the mitochondria from the ER, Ca^2+^ concentration in the mitochondria surpasses a threshold for apoptotic cell signaling, leading to MOMP and subsequently initiating apoptosis. Ceramide also induces the major ER stress response protein, activating transcription factor-6 (ATF-6), which induces apoptosis and the release of Ca^2+^ from the ER stores, thereby leading to ER-stress-mediated apoptosis [173].

VDAC located in the MOM is physically connected to IP3R in the ER through the molecular chaperone glucose-regulated protein 75 (grp75), facilitating the Ca^2+^ exchange from the ER to mitochondria [174]. Over-expression of VDAC can increase ceramide-induced Ca^2+^ levels in the mitochondrial matrix by allowing the fast diffusion of Ca^2+^ from the ER release sites to the MIM, and sensitize the cell to ceramide-induced apoptosis [120]. Ceramide-induced activation of cyclin-dependent kinase 5 (CDK5) phosphorylates microtubule-associated protein tau, which is mediated by tBid, can cluster the ER and mitochondria, facilitating Ca^2+^ transfer between the two organelles [175]. Activation of protein kinase R-like endoplasmic reticulum kinase (PERK) can also increase Ca^2+^ levels in the ER, ceramide generation via CerS6 activation, and ROS production in glioblastoma cells, while inhibition of PERK can prevent these events [176]. These mechanisms contribute to transfer Ca^2+^ from the ER to mitochondria, leading to MOMP in ceramide-induced apoptosis.

### 6.2. Regulation of Calcium Homeostasis between the ER and Mitochondria by Bcl-2 Family Proteins in Ceramide-Induced Apoptosis

Bcl-2 family members reside in the ER, where IP3R exists, can regulate ceramide-induced Ca^2+^ transfer from the ER to mitochondria. The BH3-only proteins can increase the release of Ca^2+^ from the ER, thereby increasing mitochondrial Ca^2+^ uptake, leading to apoptosis. Cells deficient for Bax and Bak have reduced resting levels of Ca^2+^ in the ER, which lowers the mitochondrial Ca^2+^ uptake, while the expression of SERCA restores the Ca^2+^ levels in the ER, thereby increasing mitochondrial Ca^2+^ uptake and restoring ceramide-induced apoptosis [177]. Oligomerized Bax/Bak inserts into the ER membrane, which leads to pore formation, thereby facilitating the release of apoptogenic proteins and possibly Ca^2+^[178]. These data suggest that Bax/Bak operate in both the ER and mitochondria, regulating mitochondrial Ca^2+^ uptake, MOMP, and ceramide-induced apoptosis.

In contrast, Bcl-2 can reduce Ca^2+^ levels in the ER, thereby inhibiting ceramide-induced mitochondrial damage and apoptosis [126]. Bcl-2 in the ER can inhibit apoptosis induced by ceramide but not by doxorubicin or TNF-α, while Bcl-xL in the ER can inhibit apoptosis induced by these agents [179]. This suggests a role of Ca^2+^ homeostasis in the ER regulated by Bcl-2 family proteins may vary with type of stimuli. In RTCs, aristolochic acid, an inhibitor of PLA2, evokes a rapid rise in intracellular Ca^2+^ levels through the release of Ca^2+^ from the ER and influx of extracellular Ca^2+^, causing the ER/mitochondria stress and apoptosis, whereas Bcl-2 can prevent these events [180]. Bcl-2/Bcl-xL may regulate Ca^2+^ homeostasis in the ER by directly interacting with IP3R and/or altering phosphorylation of the IP3R, which possibly control the channel opening [178]. Bcl-2 directly associates with and inhibits SERCA, lowering Ca^2+^ levels in the ER below the threshold level required for apoptotic signals in rat skeletal muscle cells [181]. Bcl-2/Bcl-xL also binds to IP3R, thereby inhibiting the release of Ca^2+^ from the ER [182]. Over-expression of Bcl-2 normalizes IP3R-mediated Ca^2+^ release from the ER and prevents Ca^2+^-mediated apoptosis of the cells lacking the transactivating subunit of NF-κB RelA (p65) [183]. This suggests that prevention against IP3R-mediated Ca^2+^ release from the ER by NF-κB is regulated by Bcl-2 and that IP3R in the ER functions as a pivotal target for NF-κB-mediated cell survival signaling in ceramide-induced apoptosis of embryonic fibroblasts [183]. BI-1, the ER transmembrane protein, is required for Bcl-xL-mediated lowering Ca^2+^ storage in the ER of HeLa cells [184]. Thus, these processes mediated by Bcl-2 family proteins regulate Ca^2+^ homeostasis between the ER/mitochondria, possibly regulating ceramide-induced apoptosis of RTCs.

## 7. Ceramide- and Sphingosine-1-Phosphate-Induced Cell Signaling Pathways in the Regulation of Apoptosis

### 7.1. Mitogen-Activated Protein Kinases (MAPKs) and Ceramide-Induced Apoptosis

#### 7.1.1. Ceramide-Induced Regulation of MAPKs in Apoptosis

Ceramide regulates a number of molecules involved in apoptotic pathways [2]. One of the molecular targets for ceramide is MAPKs, consisting of ERK1/2, p38MAPK and c-Jun *N*-terminal kinases (JNK1/2/3), which differentially regulate ceramide-induced apoptosis. Ceramide activates [9,167] or inhibits [185,186] ERK, and inhibition of ERK rescues [167,186], enhances [9,187] or fails to affect ceramide-induced apoptosis in other types of cells [185]. Ceramide activates [185,186], inhibits [188], or fail to affect [9] p38MAPK, and inhibition of p38MAPK rescues [185,186,189] or fail to protect [187] ceramide-induced apoptosis. Ceramide activates [9,167,185,187,189,190], inhibits [188], or fail to affect JNK [9,186], and inhibition of JNK rescues [167,187,189] or fail to protect ceramide-induced apoptosis [190]. These data suggest a crosstalk between ceramide and MAPKs in the regulation of apoptosis and that these cellular responses induced by ceramide vary with cell types and stimuli.

**Figure 5:**
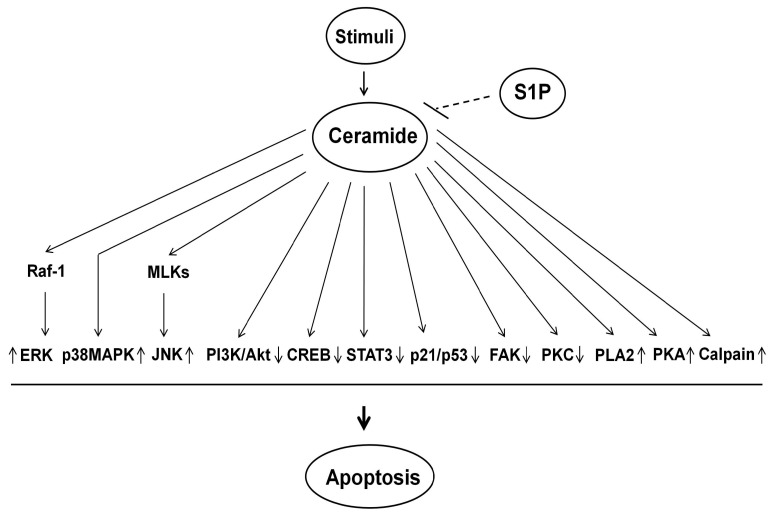
Ceramide-induced cell signaling pathways in the regulation of apoptosis of RTCs. Ceramide can regulate various cellular signaling pathways, thereby regulating apoptosis in RTCs. Ceramide activates ERK and p38MAPK, whereas JNK is activated or not activated. A role of MAPKs in the regulation of ceramide-induced apoptosis in RTCs is controversial. Ceramide can also decrease PI3K/Akt. CREB, STAT3, p21/p53, FAK, PKC, and increase PLA2, PKA and calpain, leading to apoptosis in RTCs. A crosstalk existing between MAPKs, PKC and CREB can regulate ceramide-induced apoptosis. S1P may antagonize ceramide-induced alteration of cell signaling molecules involved in apoptotic processes in RTCs. ↑, increase; ↓, decrease; ┤, suppress the expression and activity; Akt, protein kinase B; CREB, cAMP response element binding protein; ERK, extracellular signal-regulated kinase; FAK, focal adhesion kinase; MAPK, mitogen-activated protein kinase; JNK, c-Jun *N*-terminal kinase; MLKs, mixed lineage kinases; PI3K, phosphatidylinositol-3 kinase; PKA, protein kinase A; PKC, protein kinase C; PLA2, phopspholipase A2; S1P, sphingosine-1-phosphate; STAT3, signal transducer and activator of transcription-3.

Little is known about a crosstalk between ceramide and signaling molecules, including MAPKs, in the regulation of ceramide-induced apoptosis of RTCs (Figure 5). Exposure of RTCs to hydrogen peroxide, resulting in increased ceramide generation [33], can activate ERK and Akt, and inhibition of ERK protects cell death, which results in increased Akt phosphorylation [191]. The blockade of Akt potentiates hydrogen peroxide-induced apoptosis, and diminishes the protective effect conferred by ERK inhibition, suggesting a crosstalk between ERK and Akt in hydrogen peroxide-induced apoptosis of RTCs, in which ceramide is involved [191]. Ceramide renders HEK293 cells resistant to the mitogenic actions of insulin-like growth factor-I (IGF-1) by inhibiting IGF-1-induced ERK activity through interrupting the interaction of PKCε and Raf-1/ERK, suggesting that ceramide functions as an anti-mitogenic factor by limiting the ability of PKC-ε to form a signaling complex with Raf-1 and ERK in HEK 293 cells [192]. Ceramide also induces mitochondrial translocation of Bax, caspase activation, and activates ERK and p38MAPK but not JNK, and inhibition of ERK or p38MAPK fails to affect mitochondrial Bax translocation and ceramide-induced apoptosis in HK-2 cells [71]. This suggests a minor role of MAPKs for the regulation of mitochondrial Bax and activation of caspases in ceramide-induced apoptosis of RTCs. Exposure of NRK-52E cells to hypoxia, which increases ceramide generation via CerSs [31], can induces apoptosis via activation of PPA2, which may target PKCα and ERK but not Akt or Bcl-2, while JNK is not activated [50]. UV light activates A-SMase, generating ceramide, which in turn activates JNK that is required for apoptosis in both HEK293 cells and Jurkat cells [55]. Inhibition of ceramide production reduces UV-induced JNK activation in both cell lines and protects UV-induced apoptosis in HEK293 cells, but not in Jurkat cells. In addition, UV light also induces JNK activation and apoptosis of MCF-7 cells without ceramide production [53]. These data suggest that UV-induced JNK activation and apoptosis can be mediated via ceramide-dependent and -independent pathways, depending on cell types. Ceramide activate dMLK and MK3 activity that leads to JNK activation but not ERK or p38MAPK in HEK293 cells [193]. Ceramide activates stress activated protein kinase (SAPK)/JNK cascade but not ERK cascade in glomerular endothelial cells, while ceramide induces apoptosis via activation of ERK but not SAPK/JNK in glomerular mesangial cells [194]. Taken together, these data suggest that ceramide-induced MAPKs activation and its role in the regulation of apoptosis may vary with different cell types and stimuli.

Cooperation of the members of MAPKs can regulate ceramide-induced apoptosis in some types of cells [9,189]. Ceramide and paclitaxel synergistically activate ERK and JNK, leading to cell death in pancreatic cancer cells [9], Inhibition of JNK fails to inhibit ceramide, paclitaxel, or both-induced JNK and ERK activities, while inhibition of ERK both inhibits ERK and JNK, thereby enhancing cytotoxicity [9]. Ceramide activates JNK, and its inhibition alone partially rescues ceramide-induced apoptosis, but with simultaneous inhibition of p38MAPK completely blocks ceramide-induced apoptosis in neuronal cells [189]. However, ceramide fails to affect JNK activity and inhibition or activation of both ERK and p38MAPK does not affect ceramide-induced apoptosis, suggesting a minor role of crosstalk between the members of MAPKs in the regulation of ceramide-induced apoptosis of RTCs [71].

#### 7.1.2. Mechanism of Ceramide-Induced Activation of MAPKs

Ceramide can up-regulate a tumor suppressor gene, thioredoxin-interacting protein (Txnip), which activates p38MAPK and JNK, thereby leading to apoptosis in Jurkat cells [195]. Mixed lineage kinases (MLKs) are mitogen-activated protein kinase kinase kinases (MAPKKK) that activate JNK activity, leading to apoptosis. Ceramide can activate dMLK and MLK3 that in turn activate JNK in HEK293 cells, and inhibition of dMLK and MLK3 attenuates ceramide-induced JNK activation without affecting ceramide-induced activation of p38MAPK or ERK [193]. This finding suggests that MLKs are upstream of JNK in ceramide-induced apoptosis. *S*-transferase (GST)-melanoma differentiation-associated gene-7 (MDA-7), a novel cytokine, which causes plasma membrane clustering of CD95 and the association of CD95 with procaspase-8, induces ceramide accumulation via A-SMase and CerS6, inactivation of ERK, and activation of PERK, p38MAPK and JNK, thereby leading to apoptosis in renal carcinoma cells [196]. Knockdown of CD95 expression abolishes GST-MDA-7-induced phosphorylation of PERK. Knockout or expression of a dominant negative PERK restores ERK activity and inhibits activation of p38MAPK and JNK, rescuing apoptosis [196], suggesting that PERK is upstream of MAPKs. Ceramide directly binds to and activates protein kinase c-Raf, leading to subsequent activation of ERK in glomerular mesangial cells [197]. Ceramide also directly binds to and activates PKCζ, which associates with phosphorylated SAPK kinase (SEK) and MAPK kinase kinase-1 (MEKK1), forming elements of the SAPK signaling complex, resulting in subsequent growth suppression in HEK293 cells [198]. This finding suggests that ceramide enhances the ability of PKCζ to form a signaling complex with MEKK1, SEK, and SAPK.

#### 7.1.3. MAPKs Regulate the Enzymes Involved in Ceramide Metabolism

MAPKs can regulate ceramide generation by affecting the enzymes involved in sphingolipids metabolism, which in turn regulate ceramide-induced apoptosis [6,7]. JNK activates phosphorylation of N-SMase1, generating ceramide in Jurkat cells [199]. A-SMase can be activated by ERK, regulating ceramide generation and apoptosis in human melanoma cells [200]. Upon apoptotic stimuli, proteosomal turnover of CerS1 required for its activation is regulated by the opposing actions of p38MAPK (a positive regulator) and PKC (a negative regulator) in HEK293 cells [201]. In addition, turnover and translocation of CerS1 from the ER to the Golgi is regulated by PKC in HEK293 cells [39] (Table 1). In glomerular mesangial cells, N-CDase can be blocked by inhibition of p38MAPK [202]. These data suggest a potential role of MAPKs in the regulation of ceramide metabolism, thereby affecting ceramide-induced apoptosis in the kidney.

#### 7.1.4. A Crosstalk between MAPKs and Bcl-2 Family Proteins in the Regulation of Ceramide-Induced Apoptosis

A crosstalk exists between MAPKs and Bcl-2 family proteins in the regulation of ceramide-induced apoptosis. Ceramide induces mitochondrial Bax translocation and apoptosis, which are blocked by inhibition of ERK, JNK, or both in neuronal cells [167,187]. In addition, ceramide can induce apoptosis through mitochondrial Bax translocation in HL-60 cells, and p38MAPK is upstream of these events since inhibition of p38MAPK attenuates these events [203]. Over-expression of Bcl-2 inhibits ceramide-induced JNK activation, indicating that Bcl-2 is upstream of ceramide-induced JNK pathway in prostate carcinoma cells [204]. In TNF-α-induced apoptosis, which results in ceramide generation [54,59], over-expression of Bcl-xL inhibits TNF-α-induced signaling, including NF-κB, activator protein 1 (AP-1), MAPK, and JNK, and apoptosis in HL-60 cells [205]. In addition, JNK down-regulates the Bcl-2 promoter activity in β cells [206]. Furthermore, ceramide can promote phosphorylation of Bim and induce translocation of phosphorylated Bim and active JNK to the mitochondria, and the localization of these molecules is consistent, suggesting that JNK may participate in ceramide-induced apoptosis in human lung cancer cells by a mechanism involving Bim [207].

Little is known about a role of crosstalk between MAPKs and Bcl-2 family proteins in ceramide-induced apoptosis of RTCs. Ceramide enhances the mitochondrial Bax translocation and activation of ERK and p38MAPK but not JNK, whereas inhibition of ERK or p38MAPK fails to affect ceramide-induced mitochondrial Bax translocation and apoptosis, suggesting no crosstalk between Bax and MAPKs in ceramide-induced apoptosis in RTCs [71]. This issue deserves further investigations in ceramide-induced apoptosis of RTCs.

### 7.2. Signaling Molecules Other than MAPKs Regulated by Ceramide

Ceramide either directly or indirectly interacts with the Ca^2+^-dependent lipid binding C2 domain of PKCα, which in turn induces translocation of PKCα to the Golgi compartment in HEK293 cells [97]. Ceramide can induce apoptosis by inhibiting Akt and cAMP response element binding protein (CREB)-like immunoreactivities in LLC-PK1 cells [65] (Figure 5). In addition, ceramide-induced inactivation of Akt sensitizes the cells to ligand (TRAIL)-induced apoptosis in renal cell carcinoma cells [208]. Inhibition of SphK2 can also increase ceramide levels and decrease S1P levels, which in turn decreases the expression and activity of signal transducer and activator of transcription-3 (STAT3), Akt, ERK1/2, p21, p53 and focal adhesion kinase (FAK), leading to autophagy in A498 kidney adenocarcinoma cells [209].

Ceramide increases Ca^2+^ATPase activity in basolateral membranes via its phosphorylation by protein kinase A (PKA), resulting in activation of the Ca^2+^ pump, while PKC inhibits the Ca^2+^ pump [133]. However, PKC-induced inhibition of Ca^2+^ATPase activity is abolished by the ceramide-induced PKA-mediated phosphorylation, in RTCs [133], resulting in increased intracellular Ca^2+^. Ceramide binds to and differentially modulates the activity of PKCα and PKCδ but not PKCε or PKCζ in glomerular mesangial cells, which may regulate apoptosis [197]. In contrast, PKCα, PKCδ, PKCζ and PKCη are expressed in RTCs, and ceramide-induced inhibition of PKC induces apoptosis [98]. Hypoxia-induced ceramide generation [31,32] can induce activation of PP2A and up-regulation of PP2A B56α regulatory subunit as well as suppression of PKCα since PKCα negatively regulates B56α expression, leading to necrosis and apoptosis in NRK-52E cells [50]. Cadmium increases ceramide formation via CerSs, which enhances activities of calpains and caspase-3, leading to apoptosis of RTCs [60]. Oxalate activates PLA2 via ceramide generation in MDCK cells, leading to apoptosis [57]. These data suggest that various signaling molecules other than MAPKs as downstream events of ceramide play an important role in the regulation of ceramide-induced apoptosis of RTCs.

### 7.3. S1P-Induced Signaling Pathway in Apoptosis of RTCs

Intracellular S1P can inhibit ceramide-induced apoptosis through the regulation of many cellular signaling events (Figure 4 and Figure 5). S1P antagonizes apoptosis by inhibiting release of cytochrome C and Smac/DIABLO, and ROS generation from mitochondria [1]. S1P can induce up-regulation of anti-apoptotic Bcl-2/Bcl-xL and Mcl-1, and downregulation of pro-apoptotic Bax, Bad, and Bim, as well as regulate MAPKs, PI3K/Akt, and PKC, thereby preventing apoptosis in various cells [1,210,211].

Regarding a role of S1P-induced cell signaling in the regulation of renal apoptosis, low-density lipoproteins (LDL) induces expression of the pro-fibrotic connective tissue growth factor through SphK1/S1P(1)R-dependent activation of ERK and JNK in glomerular mesangial cells [212]. As described earlier, isoflurane protects oxidant-induced cytotoxicity by enhanced activation of ERK and Akt and expression of HSP70 in HK-2 cells, which are inhibited by a SphK inhibitor and S1P(1/3)R antagonist, and over-expression of SphK1 prevents oxidant-induced cytotoxicity [80]. Expression of SphK1 can prevent I/R-induced apoptosis in the kidney and RTCs through SphK1/S1P(1)R-mediated induction of HSP27 expression [84]. In contrast, over-expression of S1P lyase, which lowers intracellular levels of S1P, can enhance apoptosis in HEK293 cells via activation of p38MAPK, p53, p53-inducible death domain protein, and caspase-2 [91]. Activation of ERK can directly phosphorylate SphK1, which activates SphK1 activity and its translocation from the cytosol to the plasma membrane in HEK293 cells, leading to increased intracellular S1P levels [213]. A selective A(1)AR agonist and S1P(2)R antagonist protect I/R-induced renal injury through SphK1/S1PR-dependent nuclear translocation of HIF-1α and Rho kinase activation in RTCs [37,82]. A S1P(1)R agonist attenuates LPS- or hypoxia/reoxygenation-induced apoptosis in RTCs by activating ERK and/or Akt pathways [81]. These data suggest that S1P/S1PRs-induced signaling pathways play a protective role for ceramide-induced apoptosis of RTCs. Because the detailed review of S1P-induced cell signaling pathways in the regulation of apoptosis is beyond the scope, the readers are referred to the excellent review of S1P signaling in mammalian cells [1].

## 8. Strategy for Preventing Ceramide-Induced Apoptosis of RTCs by Growth Factors

Epidermal growth factor (EGF) can regulate apoptosis through activation of MAPKs [214]. Ceramide, paclitaxel, or both synergistically induce apoptosis and phosphorylation of ERK, JNK, and EGF receptor (EGFR) but not p38 or Akt in pancreatic cells [9]. In addition, phosphorylated EGFR, ERK and JNK are inhibited by EGFR inhibitor, and they are also blocked by ERK inhibitor but not JNK inhibitor, suggesting that the combination of paclitaxel and ceramide synergistically induce cell death through differential activation of EGFR-mediated MAPKs and that inhibitors of EGFR and ERK may further enhance the paclitaxel and ceramide effect [9]. Inhibition of EGFR and PKA can enhance apoptosis in prostatic cancer cells by increasing ceramide generation and by activating a caspase cascade in a mitochondrial-mediated manner [215]. Proliferation of smooth muscle cells induced by oxidized LDL (oxLDL) involves the SM/ceramide/S1P pathway, which leads to ERK activation and DNA synthesis, and the EGFR/PI3K/Akt pathway can prevent the apoptotic effect of oxLDL [216]. C2-ceramide and PLA2 inhibits EGF-induced activation of EGFR, which is associated with arachidonic acid release and an increase in intracellular ceramide formation, leading to inhibition of proliferation in human epidermoid carcinoma cells [217]. CD95 ligand (CD95L)-induced apoptosis involves A-SMase- and PKCζ-dependent activation of NADPH oxidase isoforms, which are required for Yes/EGFR/CD95 interactions as upstream events of CD95 activation in rat hepatocytes [218]. These lines of evidence suggest that EGF/EGFR may prevent ceramide-induced apoptosis through MAPKs-dependent and -independent pathways.

Growth factors can also activate SphKs and induce S1P generation, which in turn inhibits apoptosis. Growth factors, including platelet-derived growth factor (PDGF), vascular endothelial growth factor (VEGF), nerve growth factor (NGF), and EGF, can activate SphK1, resulting in increased intracellular S1P levels [1]. Binding of EGF to EGFR enhances activities of JNK, p38MAPK and SphK1 pathways, but JNK- and SphK1-dependent pathways, but not p38MAPK-dependent pathways prevents apoptosis in cytotrophoblasts [219]. In addition, inhibition of SphK1 does not affect EGF-stimulated phosphorylation of PI3/Akt, ERK or p38MAPK, but inhibition of PI3/Akt inhibits the EGF-stimulated increase in SphK1 activity [219]. EGF activates and translocates SphK1 to the plasma membrane in MCF-7 cells, and down-regulation of SphK1 can reduce EGF-stimulated cell growth and sensitizes the cells to apoptotic stimuli [220]. S1P activates EGFR, PDGF receptor (PDGFR), p38MAPK, SAPK/JNK, intercellular adhesion molecule-1 (ICAM-1), vascular cell adhesion molecule-1 (VCAM-1), and cyclooxygenase-2 (COX-2), and S1P/S1P(1)R functions as proinflammatory signaling pathways through EGFR and PDGFR transactivation in vascular smooth muscle cells [221].

In RTCs, binding of EGF to EGFR stimulates activities of ERK, phospholipase C (PLC), and SphK, resulting in increased intracellular levels of S1P, while the EGF/EGFR-mediated Ca^2+^ mobilization, which may regulate apoptosis, requires activation of PLC and SphK in HEK293 cells [222]. On the other hand, EGF activates both ERK and p38MAPK, but not JNK in HK-2 cells [223], while EGF rescues ceramide-induced apoptosis in MAPKs-independent pathway without affecting mitochondrial translocation of Bax [71]. These data suggest an anti-apoptotic role of EGF possibly through PLC- and S1P-dependent pathways for ceramide-induced apoptosis in RTCs.

IGF can also regulate SphKs activity. IGF-II binding to the IGF-II/mannose-6-phosphate (M6P) receptor activates ERK pathway by triggering SphK1-dependent transactivation of G protein-coupled S1P receptors in HEK293 cells [224]. This study also shows that inhibition of PKCβ2 and PLC abolishes IGF-II-stimulated translocation of SphK1 to the plasma membrane and activation of SphK1, suggesting that PKCβ2/PLC are upstream regulators of SphK, leading to suppression of apoptosis. Ceramide inhibits ERK, PKC-ε, and IGF-1-induced cell growth by limiting the IGF-1-induced ability to form a signaling complex of PKC-ε with Raf-1/ERK in HEK293 cells [192]. A radiocontrast, ioversol, induces ceramide generation via CerSs and apoptosis, through inhibition of IGF-1-dependent Akt activation in LLC-PK1 cells [65]. In addition, PDGF can increase S1P generation and decrease sphingosine levels, thereby regulating cell cycle in HEK293 cells [225]. Furthermore, the tyrosine residue of PDGFR responsible for binding of PLCγ and Ca^2+^ mobilization of downstream of PLCγ appear to be required for PDGF-induced activation of SphK in TRMP canine kidney epithelial cells [226]. Taken together, these data suggest that SphKs are activated by growth factors, thereby decreasing ceramide/S1P ratio and preventing ceramide-induced apoptosis. Thus, growth factors may be a potential therapeutic target for ceramide-induced apoptosis in RTCs.

## 9. Conclusions and Future Perspective 

Ceramide induces apoptosis, and S1P functions as a survival factor in various cells. In this review, I summarized and discussed current evidence for a role of ceramide and the balance between ceramide and S1P in the regulation of apoptosis in RTCs. Mitochondria play a central role in the regulation of ceramide-induced apoptosis through its function, which is tightly regulated by Bcl-2 family proteins. Anti-apoptotic Bcl-2 members (e.g., Bcl-2/Bcl-xL) can maintain mitochondrial integrity, while pro-apoptotic Bcl-2 members (e.g., Bax/Bak) induce MOMP. Bcl-2 family repertoires can regulate at least the formation of MAC, ceramide channel, VDAC, and MPT pore, leading to MOMP. As a result, MOMP-mediated downstream events, including ROS generation, MAPKs, other protein kinases and signaling molecules, the release of cytochrome C into the cytosol and activation of a caspase cascade, can initiate apoptotic process. Anti-apoptotic Bcl-2 family members may reduce ceramide accumulation, formation of MAC, ceramide channel, VDAC, and MPT pore opening, as well as increase intracellular S1P levels by affecting the enzymes involved in ceramide metabolism. In contrast, pro-apoptotic Bcl-2 family members have the opposite effects, resulting in increased ceramide generation, formation of MAC, ceramide channel, VDAC and MPT pore opening, and decreased S1P levels. On the other hand, growth factors may prevent ceramide-induced apoptosis possibly through MAPKs-dependent and -independent pathways (e.g., S1P- and PLC-dependent pathways), and may be a potential therapeutic strategy for prevention of ceramide-induced apoptosis in RTCs. Currently, very little is known about a role of ceramide and other sphingolipids in the regulation of apoptosis and its modulation by growth factors in the kidney. For better understanding of a role of ceramide and other sphingolipids, especially S1P, in kidney diseases, extensive studies should focus on the precise mechanism(s) by which they function as pro-apoptotic or survival factors. This will allow us to establish the novel therapeutic strategies, including targeting the Bcl-2 family proteins, modalities of regulating the enzymes involved in ceramide metabolism, and growth factors for prevention of ceramide-induced apoptosis of RTCs.

## Abbreviations

ACEC: apical ceramide-enriched compartment
AIF: apoptosis inducing factor
ANT: adenine nucleotide translocase
AP-1: activator protein 1
APF-1: apoptosis protease-activating factor-1
AR: adenosine receptor
ATP: adenosine 5'-triphosphate
BI-1: Bax inhibitor-1
BMK: baby mouse kidney
CDases: ceramidases
CDK5: cyclin-dependent kinase 5
CerSs: ceramide synthases
CERT: ceramide transfer protein
C1P: ceramide-1-phosphate
CREB: cAMP response element binding protein
Δψm: mitochondrial membrane potential
Drp1: dynamin-related protein 1
EGF: epidermal growth factor
EGFR: EGF receptor
ER: endoplasmic reticulum
ERK: extracellular signal-regulated kinase
FAK: focal adhesion kinase
GSH: glutathione
GSLs: glycosphingolipids
GSSG: oxidized GSH
HEK: human embryonic kidney
HIF: hypoxia-inducible factor
HSP: heat shock protein
IAP: inhibitor of apoptosis
IGF-1: insulin-like growth factor-1
IGF-II: insulin-like growth factor-II
IP3R: 1,4,5-trisphosphate receptor
IL: interleukin
I/R: ischemia/reperfusion
JNK: c-Jun *N*-terminal kinases
3-keto-dihydro-Sph: 3-keto-dihydrosphingosine
LPS: lipopolysaccharide
MAMs: mitochondrial-associated membranes
MAPKKK: mitogen-activated protein kinase kinase kinases
MAPKs: mitogen-activated protein kinases
MDCK: Madin-Dabry canine kidney
MEKK: MAP/ERK kinase kinase
MIM: mitochondrial inner membrane
MLKs: mixed linage kinases
MOM: mitochondrial outer membrane
MOMP: mitochondrial outer membrane permeability
MPT: mitochondrial permeability transition
PDGF: platelet-derived growth factor
PDGFR: PDGF receptor
PERK: protein kinase R-like endoplasmic reticulum kinase
PI3K: phosphatidylinositol 3-kinase
PKA: protein kinase A
PKC: protein kinase C
PLA2: phospholipase A2
PLC: phospholipase C
PP2A: protein phosphatase 2A
ROS: reactive oxygen species
RTCs: renal tubular cells
SAPK: stress activated protein kinase
SERCA: sarcoplasmic-endoplasmic reticulum Ca^2+^-ATPase
SM: sphingomyelin
SMases: sphingomyelinases
SMSs: SM synthases
SphKs: sphingosine kinases
S1P: sphingosine-1 phosphate
S1PRs: S1P receptors
SPT: serine palmitoyl transferase
STAT: signal transducer and activator of transcription
tBid: truncated Bid
TNF: tumor necrosis factor
UV: ultraviolet
VDAC: voltage-dependent anion channel
